# Reporting of harms in oncological clinical study reports submitted to the European Medicines Agency compared to trial registries and publications—a methodological review

**DOI:** 10.1186/s12916-021-01955-0

**Published:** 2021-04-08

**Authors:** Asger S. Paludan-Müller, Perrine Créquit, Isabelle Boutron

**Affiliations:** 1 Centre for Evidence-Based Medicine Odense (CEBMO) and Cochrane Denmark , Department of Clinical Research, University of Southern Denmark, JB Winsløwsvej 9b, 3rd Floor, 5000 Odence C, Denmark; 2 Open Patient data Exploratory Network (OPEN) , Odense University Hospital , Odense, Denmark; 3 Direction de la recherche Clinique, Hôpital Foch, Suresnes, France; 4 Université de Paris, CRESS, INSERM, INRA, F-75004 Paris, France; 5 Cochrane France, Paris, France; 6 Centre d’Epidémiologie Clinique, AP-HP (Assistance Publique des Hôpitaux de Paris), Hôpital Hôtel Dieu, Paris, France

**Keywords:** Systematic reviews, Reporting bias, Clinical study reports, Adverse events, Harms, Registries

## Abstract

**Background:**

An accurate and comprehensive assessment of harms is a fundamental part of an accurate weighing of benefits and harms of an intervention when making treatment decisions; however, harms are known to be underreported in journal publications. Therefore, we sought to compare the completeness of reporting of harm data, discrepancies in harm data reported, and the delay to access results of oncological clinical trials between three sources: clinical study reports (CSRs), clinical trial registries and journal publications.

**Methods:**

We used the EMA clinical data website to identify all trials submitted to the EMA between 2015 and 2018. We retrieved all CSRs and included all phase II, II/III or III randomised controlled trials (RCTs) assessing targeted therapy and immunotherapy for cancer. We then identified related records in clinical trial registries and journals. We extracted harms data for eight pre-specified variables and determined the completeness of reporting of harm data in each of the three sources.

**Results:**

We identified 42 RCTs evaluating 13 different drugs. Results were available on the EMA website in CSRs for 37 (88%) RCTs, ClinicalTrials.gov for 36 (86%), the European Clinical Trials Register (EUCTR) for 20 (48%) and in journal publications for 32 (76%). Harms reporting was more complete in CSRs than other sources. We identified marked discrepancies in harms data between sources, e.g. the number of patients discontinuing due to adverse events differed in CSRs and clinical trial registers for 88% of trials with data in both sources. For CSRs and publications, the corresponding number was 90%. The median (interquartile range) delay between the primary trial completion date and access to results was 4.34 (3.09–7.22) years for CSRs, 2.94 (1.16–4.52) years for ClinicalTrials.gov, 5.39 (4.18–7.33) years for EUCTR and 2.15 (0.64–5.04) years for publications.

**Conclusions:**

Harms of recently approved oncological drugs were reported more frequently and in more detail in CSRs than in trial registries and journal publications. Systematic reviews seeking to address harms of oncological treatments should ideally use CSRs as the primary source of data; however, due to problems with access, this is currently not feasible.

**Supplementary Information:**

The online version contains supplementary material available at 10.1186/s12916-021-01955-0.

## Background

Systematic reviews of randomised controlled trials (RCTs) are considered the gold standard for evaluating the effectiveness and harms of interventions [1]. However, results of many completed RCTs are not published, which leads to reduced power and potential publication bias in reviews [2–4]. Moreover, peer-reviewed publications are not always an accurate reflection of how trials were planned, conducted and analysed. A lack of transparency or missing information on harms is common [3, 5].

One potential source of unpublished data is clinical study reports (CSRs): extensive reports prepared by pharmaceutical companies and submitted to regulatory authorities as a part of an application for marketing authorisation. The structure of CSRs is outlined in a guideline from the International Conference on Harmonisations [6]. Access to CSRs has historically been difficult [7], but since 2015, the European Medicines Agency (EMA) has launched an initiative (policy 0700) to increase transparency of information on medicinal drugs by providing access to CSRs submitted to the agency. However, the EMA has not published any CSRs since December 4, 2018, when the initiative was paused indefinitely during the EMA’s move to Amsterdam [8]. Although several systematic reviews have included CSRs [9–12] and a questionnaire study found that respondents consider CSRs valuable for systematic reviews [13], a 2014 study found that most systematic reviews continue to rely on publications as the primary source of data [14].

Several studies have compared reporting in publications, trial registries and CSRs; for example, a study found that CSRs had higher reporting quality than did registry reports and publications [15], a finding that was confirmed in several other studies [16–20]. However, no study has systematically compared reporting of harms in trial registries and publications with a large sample of recent CSRs from oncological trials.

Targeted therapy and immunotherapy for cancer have revolutionised the care of most patients with cancer. Several of these specific oncologic drugs have recently been approved by the US Food and Drug Administration and EMA. Evaluating the harms of these new drugs is essential. Thus, we aimed to compare the delay to access results of oncological RCTs, the completeness of reporting harm data and discrepancies in harm data reported between the three sources: CSRs available on the EMA Website, clinical trial registries and journal publications.

## Methods

We identified all trials evaluating targeted therapy and immunotherapy for cancer available on the EMAs clinical data Website and retrieved the related CSRs. Then, we systematically searched for the related records in clinical trial registries and related publications. Finally, we compared the delay to access results, the completeness of reporting of harm data and discrepancies in harm data reported between the three sources.

### Identification of trials

In November 2019, we used the EMA’s clinical data website (https://clinicaldata.ema.europa.eu) to identify all submissions for marketing authorisation or extension of indication under the EMA policy 0070. We updated the search in June 2020 and identified no new submissions. For all submissions, we extracted the product name, active substance, marketing authorisation holder and Anatomical Therapeutic Chemical (ATC) code. We selected the ATC codes for monoclonal antibodies (L01XC) and protein kinase inhibitors (L01XE) corresponding to targeted therapy and immunotherapy.

Once we had identified all eligible active substances, we downloaded all documents from the EMA website (i.e., CSRs and related documents) and used these to create a list of all trials submitted to the EMA. We included phase II, II/III or III RCTs that were part of a submission for a targeted therapy or immunotherapy. We excluded trials that compared only different dosages of the same treatment.

Two reviewers (ASP-M and PC) independently identified trials from the documents for one-quarter of the eligible active substances. Because of no discrepancies in this identification, the remaining identifications involved one reviewer (ASP-M).

### Identification of related clinical-trial registry records for the identified RCTs

One reviewer (ASP-M) systematically searched ClinicalTrials.gov and the European Clinical Trials Register (EUCTR) by using (1) the trial registry identifier or ID number if mentioned in the CSR or (2) the name of the experimental drug (or its international non-proprietary name). If we were still unable to identify the corresponding trial, we used other keywords (e.g. treatment comparator and indication). The records identified were systematically checked by a second reviewer (PC). Then we checked whether results were posted on the trial registries identified. If the study was registered in both registries, we extracted data from both.

### Identification of results publications for identified RCTs

We first searched for citations listed in trial registries. For ClinicalTrials.gov, the only registry to give access to citations, we used the citations listed under “publication of results” and “publications automatically indexed to this study by ClinicalTrials.gov identifier (NCT Number)”. We included all publications reporting results for the trial identified. We did not include publications of reviews or publications that presented pooled analyses of several trials and did not include data from the individual trial. If no publications were indexed in the registry record, we searched MEDLINE and EMBASE by using the name of the experimental drug, treatment comparator, indication and name of the principal investigator.

### Data extraction

For each trial, we extracted information from the CSR available on the EMA Website, the clinical trial registry records (both ClinicalTrials.gov and EUCTR) and all related publications. The extracted information was entered in a data extraction spreadsheet. Two reviewers (ASP and PC) independently extracted data for 10% of trials. Because of only minor disagreements, one reviewer (ASP-M) extracted the data for the remaining trials. All extractions were then checked by a second reviewer (PC). All discrepancies were resolved by discussion.

We extracted the following information for each trial:

1. *General characteristics*: name of trial, name of studied drug, clinical development phase, condition; number of centres, number of arms, number of participants randomised, whether the trial was a non-inferiority trial, the primary outcome, funding and whether the trial was blinded.
2. *Delay in access to trial results:* The CSRs included in this project are released under the EMA policy 0070 [21], which dictates that clinical data submitted to the EMA as part of a marketing authorisation application or a post-authorisation procedure shall be released once the concerned procedure (hereafter EMA procedure) has been finalised. We recorded the date of finalisation of the procedure for all included submissions by using the European Commission’s register [22] and determined the delay between the finalisation of the procedure and publication of CSRs on the EMA website.

To determine the time between completion of the study and release of results in each source, we recorded the primary trial completion date (i.e. the date of the last participant’s final follow-up visit for measurement of the primary outcome) from ClinicalTrials.gov. If this was not available, we checked the other sources (CSRs, publications, and EUCTR) for a primary completion date. We also recorded for each source the date when the results were released and available. For trials with multiple publications, we used the earliest publication date. We then calculated the delay between primary trial completion date and availability of results for each source.

3) *Completeness of reporting harm data and discrepancies in harm data*: We extracted the following information from all three sources of data for each trial: number of patients randomised, whether a definition of safety population was provided, number of patients in the safety population, threshold for reporting adverse events (e.g. 10%, 5% or none), number of patients experiencing at least one adverse event, total number of adverse events, number of patients experiencing at least one serious adverse event, total number of serious adverse events, number of patients experiencing at least one adverse event judged to be grade 3–5 according to the Common Terminology Criteria for Adverse Events (CTCAE), total number of adverse events judged to be grade 3–5 according to the CTCAE, number of patients discontinuing the trial due to adverse events, number of deaths due to adverse events, and whether a description of the process of determining whether a death was due to adverse events, including whether the person(s) making the judgement were blinded, was provided. For all variables, we recorded the numbers per arm. Some sources reported CTCAE grade 3–4 events rather than grade 3–5 events. If the number of grade 5 events was reported separately, we added the numbers. If the number of grade 5 events was not reported, we still gave the trial a “yes” for the question, extracted the number of grade 3–4 events and noted this.

### Analysis

We compared reporting of the different variables defined above in CSRs with that in clinical trial registries and publications, separately. We performed Kaplan-Meier analysis on the delay from primary trial completion date to the publication of the CSR, the first publication of results in trial registries, and a publication in a medical journal. If a trial had not been published in a source, we calculated the delay between the primary completion date and June 29, 2020 and considered the trial right censored. For numerical variables reported in at least two of the data sources, we examined whether the numbers reported were the same. For this analysis, we pooled results from ClinicalTrials.gov and EUCTR. If results were available from both registries, we used the data from ClinicalTrials.gov for the analysis of discrepancies.

### Patient involvement

No patients were involved in the planning or conduct of this study.

## Results

### Selection of trials

**Fig. 1:**
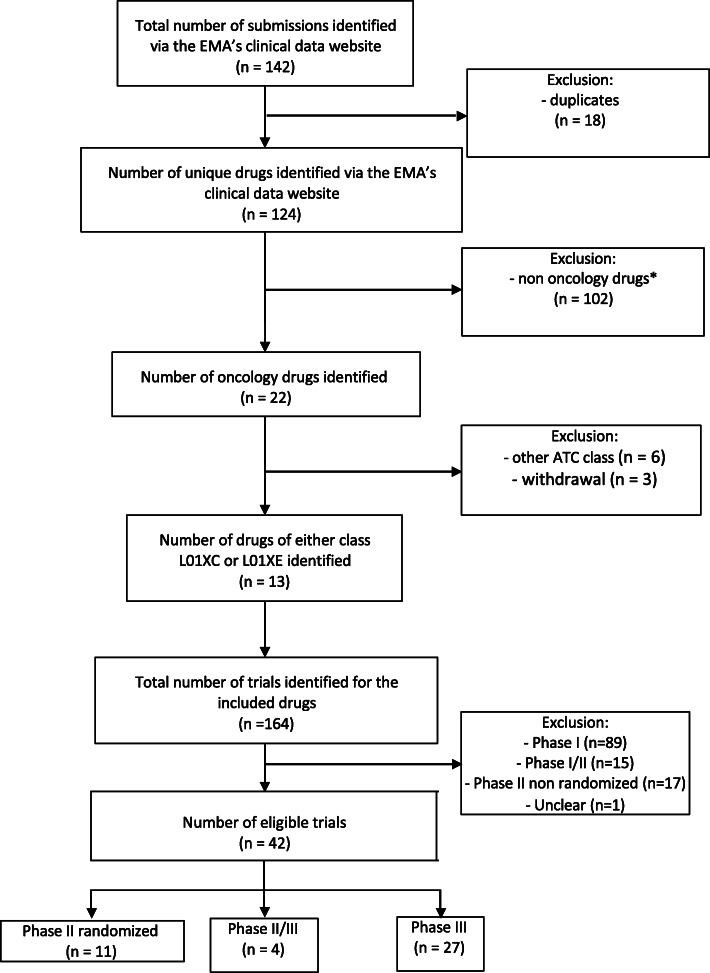
Flowchart of inclusion of trials

We identified 142 submissions through the EMA clinical data Website. These submissions corresponded to 124 unique substances: 22 concerned oncology drugs and 13 of these corresponded to targeted therapy and immunotherapy (Additional file 1). For these 13 drugs included in the study, we identified 164 unique trials, of which 42 met our eligibility criteria (phase II, II/III or III RCTs). The inclusion process is shown in Fig. 1.

The drugs included and the number of trials for each drug are in Table 1. The median number of randomised patients in the included trials was 364 (range 142–666) (Table 2). The primary outcome was progression-free survival for 27 of the 42 (64%) included trials, overall survival for eight (19%) and both for three (7%). The remaining four trials (7%) had other primary outcomes. Additional characteristics of included studies are in the Additional file 1: Table S1. Publications matched to individual trials are presented in Additional file 1: Table S2 [23–112].

**Table 1:** Included drugs and trials

Drug name	Number of trials	Pharmaceutical company	Type of cancer	Trial name available
Afatinib	6	Boehringer Ingelheim	Head and neck squamous cell carcinoma, non-small cell lung cancer	LUX-Head and Neck 1, LUX-Lung 5, LUX-Lung 6, LUX-Lung 8, LUX-LUNG 1
Bevacizumab	6	Roche	Non-small cell lung cancer	ATLAS, EURTAC, BeTa
Cabozantinib	4	Exelixis	Medullary thyroid cancer, renal cell carcinoma, prostate cancer	EXAM, METEOR, COMET-1, COMET-2
Cediranib	5	AstraZeneca	Ovarian cancer, colorectal cancer, renal cell carcinoma, glioblastoma	ICON6, HORIZON III, HORIZON II, REGAL
Cediranib	2	NCIC Clinical Trials Group	Non-small cell lung cancer	
Erlotinib	1	Roche	Non-small cell lung cancer	
Everolimus	1	Novartis	Neuro-endocrine tumour (gastro-intestinal or lung origin)	RADIANT-4
Lenvatinib	4	Eisai	Non-small cell lung cancer, glioma, differentiated thyroid cancer, hepatocellular carcinoma	SELECT
Nivolumab	4	Bristol-Myers Squibb	Renal cell carcinoma, non-small cell lung cancer, melanoma	CheckMate 025, CheckMate 057, CheckMate 067, Checkmate 069
Olaratumab	3	ImClone Systems	Ovarian cancer, non-small cell lung cancer, prostate cancer	
Palbociclib	3	Pfizer	Breast cancer	PALOMA-2, PALOMA-3, PALOMA-4
Pembrolizumab	3	Merck	Melanoma, non-small cell lung cancer	Keynote-006, Keynote-010, Keynote-024

**Table 2:** Characteristics of included trials

**Clinical development phase**	***N*** **(%)**
Phase II	12 (29)
Phase II/III	4 (10)
Phase III	26 (61)
**Blinding**	***N*** **(%)**
Open label	22 (52)
Double blind	20 (48)
**Primary outcome**	***N*** **(%)**
Progression-free survival	27 (64)
Overall survival	8 (19)
Progression-free survival and overall survival	3 (7)
Other	4 (7)
**Superiority design**	40 (95%)
	**Median (IQR)**
Number of participants randomised	364 (142–666)
Number of centres	99 (42–142)

*IQR* interquartile range

### Availability of sources

The EMA’s Website had complete CSRs for 37 of the 42 (88%) included trials. For the remaining five (12%) trials, the EMA Website did not contain full CSRs and only documents such as summaries, pharmacokinetic data or periodic safety reports were available; the EMA Website did not explain why full CSRs are not available for these trials, but three trials were ongoing at the time of the application for marketing authorisation. Among the 42 included trials, trial results were posted on ClinicalTrials.gov for 36 (86%) and on the EUCTR for 20 (48%). We were able to identify publications with results for 32 of the 42 (76%) included trials (all included publications are in the Additional file 1). Trial results were available in the three sources for 25 (60%) trials and in two sources for 13 (31%) (i.e. CSR and clinical trial registry for six trials, in CSR and publication for three trials, in clinical trial registry and publications for four trials). Results were available in only one source for three (7%) trials (two in CSRs and one in a clinical trial registry) and one (2%) trial had no results available.

### Delay in access to trial results

**Fig. 2:**
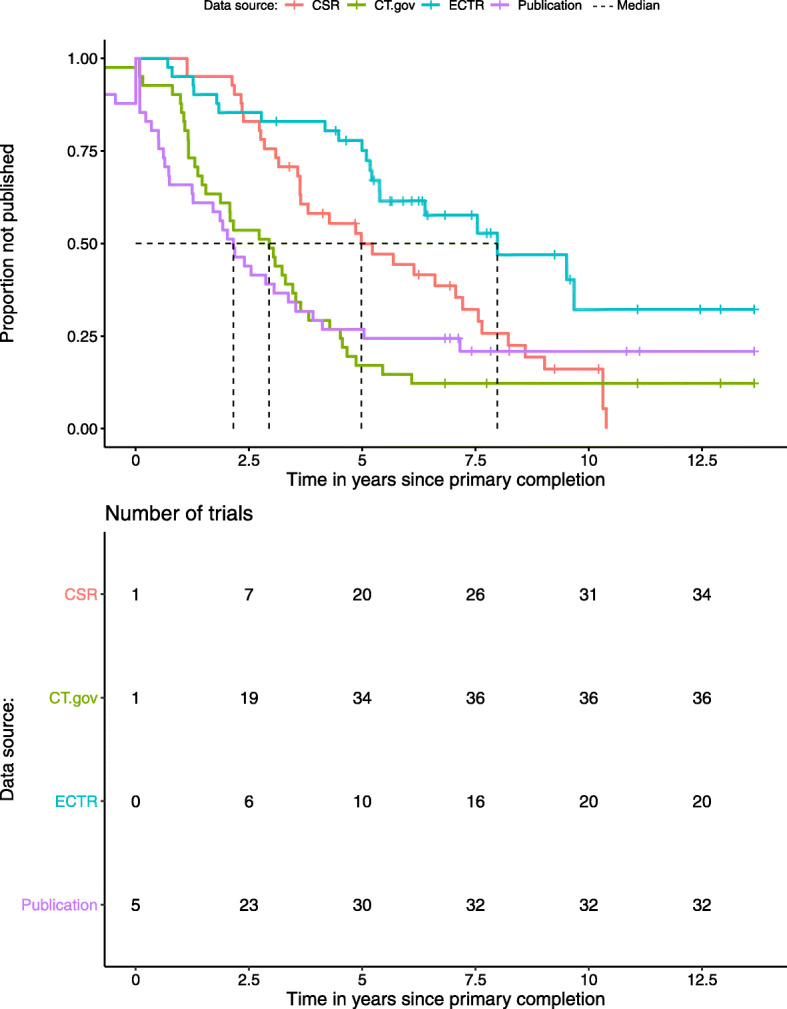
Delay between primary trial completion day and availability of results from sources in days. CSR, clinical study report; CT, ClinicalTrials.gov; ECTR**,** European Clinical Trials Register

The median delay between finalisation of the EMA procedure and availability of CSRs was 1.21 years (range 0.91–1.78). The median (interquartile range) delay between primary completion data and result availability was 4.34 (3.09–7.22) years for CSRs, 2.94 (1.16–4.52) years for ClinicalTrials.gov, 5.39 (4.18–7.33) years for the EUCTR and 2.15 (0.64–5.04) years for publications. Figure 2 shows Kaplan-Meier curves for the delay between primary trial completion dates and publication of the different sources.

### Reports of harms in each source

Table 3 shows the proportion of trials for which we could obtain data on our pre-specified variables from each of the four sources of data. For most variables, results were more frequently reported in CSRs than both trial registries and publications. The number of patients with at least one serious adverse event was reported for all trials in both CSRs and ClinicalTrials.gov and in 19/20 (95%) trials in the EUCTR but only 16/32 (50%) trials with publications. The number of patients with any adverse events was reported in all CSRs but was not available for any trials or registries because the number of patients with serious and non-serious adverse events are reported separately. The number of patients with any adverse events was available for only 13/32 (41%) trials with publications. The number of patients with CTCAE grade 3–5 events was available in 36/37 (97%) CSRs but only 14/32 (44%) publications. The CTCAE grade was not reported in either of the trial registries.

**Table 3:** Proportion of trials for which data, including harms data, could be obtained from the sources examined (*n* = 42)

	CSR	ClincalTrials.gov	EU Clinical Trials Register	Publications
Source of data identified	37 (88%)	36 (86%)	20 (48%)	32 (76%)
**Reporting of**				
Included participants				
Number of participants randomised	37 (100%)	36 (100%)	19 (95%)	32 (100%)
Number of participants in safety population	37 (100%)	36 (100%)	19 (95%)	32 (100%)
Serious adverse events (SAEs)				
Number of patients with at least one SAE	37 (100%)	36 (100%)	19 (95%)	16 (50%)
Total number of SAEs	9 (24%)	10 (28%)	17 (85%)	1 (3%)
Any adverse events (AEs)				
Number of patients with at least one AE	37 (100%)	0 (0%)	0 (0%)	13 (41%)
Total number of AEs	12 (32%)	10 (28%)	17 (85%)	0 (0%)
CTCAE grade 3–5 AEs				
Number of patients with at least one Grade 3–5 AE	36 (97%)	0 (0%)	0 (0%)	14 (44%)
Total number of Grade 3–5 AEs	6 (16%)	0 (0%)	0 (0%)	0 (0%)
Deaths due to AEs				
Number of deaths due to AEs	34 (92%)	0 (0%)	15 (75%)	12 (38%)
Information on how it was decided whether a death was considered due to an AE	10 (27%)	0 (0%)	0 (0%)	0 (0%)
Discontinuations due to AEs				
Number of patients who discontinued trial due to AEs	32 (86%)	28 (78%)	17 (85%)	25 (78%)

*CSR* clinical study report, *CTCAE* Common Terminology Criteria for Adverse Events

The total number of serious adverse events, any adverse events, and CTCAE grade 3–5 events was available in 9/37 (24%), 12/37 (32%) and 6/37 (16%) CSRs, respectively; 10/36 (28%), 10/36 (28%) and 0/36 (0%) records at ClinicalTrials.gov; and 17/20 (85%), 17/20 (85%) and 0/20 (0%) records at the EUCTR. For publications, only 1/32 (3%) reports gave the total number of serious adverse events. The number of total adverse events and grade 3–5 adverse events was not available in publications for any trial.

The number of deaths due to adverse events was available in CSRs for 34/37 (92%) trials, from ClinicalTrials.gov for no trials, from EUCTR for 15/20 trials (75%) and from publications for 12/32 (38%) trials. Only 10/37 (27%) trials in CSRs and no trials in other sources had a detailed explanation of how it was decided whether a death was due to an adverse event or progressive disease.

### Discrepancies between sources

**Fig. 3:**
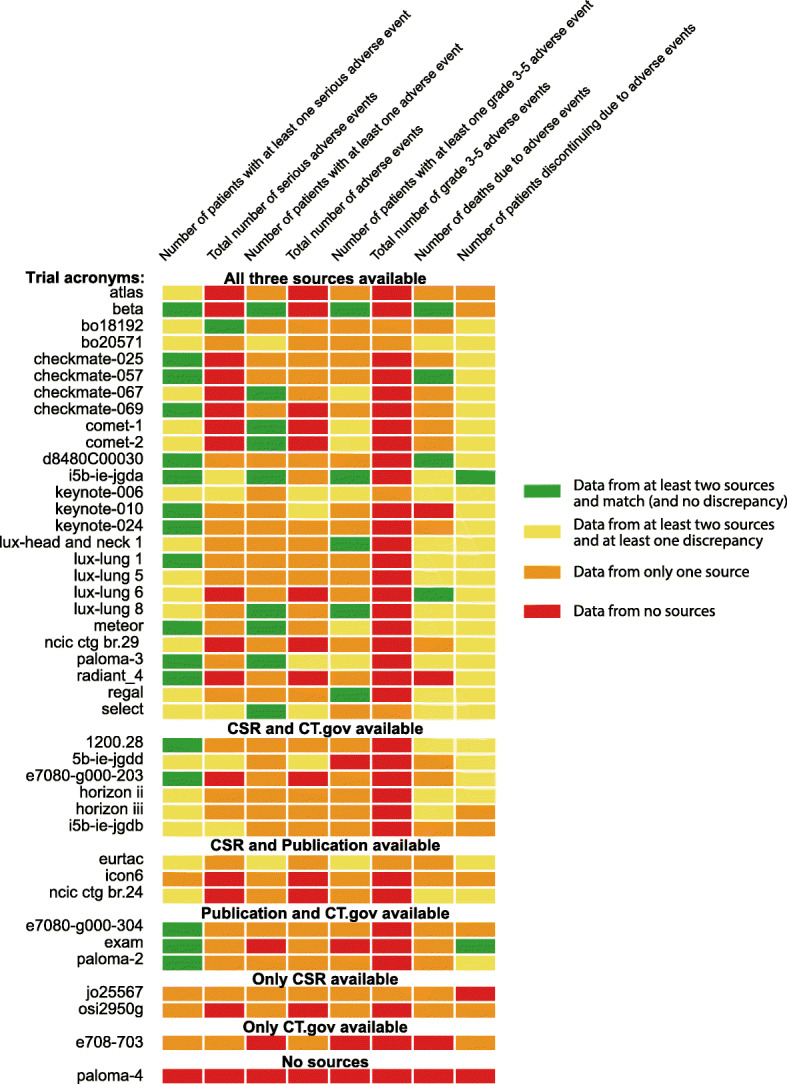
Overview of reporting and whether data matched for each trial and each variable for CSRs, publications and registries

For trials for which results were available for a variable in a minimum of two sources of data, we compared the data and noted any discrepancies. The proportion of trials with discrepancies are in Table 4. Figure 3 shows discrepancies for each variable in each included trial.

**Table 4:** Discrepancies in harms data between CSRs, trial registries, and publications for variables that were reported in two sources

	CSR and trial registries	CSR and publications	Publications and trial registries
Discrepancies			
Number of patients with at least one SAE	15/32 trials (47%)	5/13 trials (38%)	8/14 trials (57%)
Total number of SAEs	5/5 trials (100%)	0/1 trial (0%)	No trials with data from both sources
Number of patients with at least one AE	No trials with data from both sources	2/11 trials (18%)	No trials with data from both sources
Total number of AEs	5/5 trials (100%)	No trials with data from both sources	No trials with data from both sources
Number of patients with at least one Grade 3–5 AE	No trials with data from both sources	7/12 trials (58%)	No trials with data from both sources
Total number of Grade 3–5 AEs	No trials with data from both sources	No trials with data from both sources	No trials with data from both sources
Number of deaths due to AEs	12/13 trials (92%)	4/10 trials (40%)	4/4 trials (100%)
Number of patients who discontinued trial due to AEs	23/26 trials (88%)	18/20 trials (90%)	11/18 trials (61%)

*CSR* clinical study report, *AE* adverse event, *SAE* serious adverse event

We found marked discrepancies in harms data between CSRs, trial registry entries and publications. The number of patients who discontinued the treatment due to adverse events was particularly problematic, with discrepancies in 88% and 90% of trials for CSRs as compared with registries and publications, respectively. The number of deaths due to adverse events differed between CSRs and clinical trial registers in 92% of trials with data from both sources, the corresponding number was 40% for CSRs and publications. Similarly, the total number of serious adverse events differed between CSRs and clinical trial registers for all five trials whereas the numbers matched in the single trial that had data in both a CSR and a publication.

## Discussion

Our study shows that data on harms in RCTs evaluating targeted therapy and immunotherapy for cancer are reported more frequently and in more detail in CSRs than in registries and publications. However, reporting is not perfect. CSRs were missing for five (12%) trials. Three of these trials were ongoing at the time of submission of documents to the EMA which might explain the missing CSRs. The two remaining trials were completed before the submission and it is unclear why CSRs were missing for these trials. Furthermore, important data were incompletely reported; for example, the total number of serious adverse events and all adverse events was available in only 9/37 (24%) and 12/37 (32%) CSRs. Although data should be available at the date the EMA procedure is completed, we showed a median of 1.21 years between the finalisation of the procedure and publication of CSRs on the website. Additionally, the delay from primary completion of a trial until results were available was longer for CSRs than for other sources. We also demonstrated discrepancies in harms data between CSRs and other sources. One possible explanation for such discrepancies could be different selection criteria for which events to include in which reports, e.g., clinicaltrials.gov use a 5% threshold for non-serious adverse events and similar thresholds are also commonly used in journal publications. Routinely reporting all events, without thresholds, would both improve reporting and potentially solve some discrepancies.

Our results are consistent with other findings. In 2013, Wieseler et al. examined a sample of 86 trials with both a CSR and a publicly available source of data and found that serious adverse events, adverse events and withdrawals due to adverse events were more frequently reported in CSRs than another source [16]. In 2014, Maund et al. found that for nine antidepressant trials, CSRs were a more reliable source of information on harms than were journal articles [17]. In 2016, two reports described the reporting of harms of orlistat in CSRs and journal publications: both concluded that reporting of harms was more extensive in CSRs than in journal publications [18, 19]. In 2019, a study compared six CSRs for gabapentin and two for quetiapine with publications and found that in CSRs all adverse events were reported, whereas no publications reported all adverse events [20].

Our finding of non-publication of RCTs and poor reporting of harms in publications is also in accordance with previous findings [113]. The CONSORT statement outlines items that should be reported in journal publications describing RCTs and is endorsed by 585 journals [114]. However, the CONSORT statement only has one item addressing harms. An extension for harms exists and some of the items outlined in this extension are the number of participants discontinued due to harms, the frequency of all adverse events with separate information about the severity, and the number of both affected participants and the number of events [115]. Unfortunately, to our knowledge, submission of the CONSORT harms extension is not mandated by any journal, and of the *Lance*t, *New England Journal of Medicine*, *BMJ* and *JAMA*, only the *Lancet* makes specific reference to the extension in their guidance to authors [116]. Had the extension been endorsed by the journals included in this study, and thus been followed, publications would likely have fared much better.

The substantial delay between completion of trials and availability of CSRs is an important barrier to access to all data. Several teams have highlighted the need to access trial results through posting on trial registries. For example, a report from TranspariMED states that in a 2013 cohort of cancer drug trials, two thirds of trials had not posted results to ClinicalTrials.gov 3 years after completion [117]. Interventions have been proposed to improve access to results [118] and similar strategies to increase access and reduce delays for CSRs should be developed.

Our study is the first to compare reporting of harms in CSRs released under EMA policy 0070 with publications and trial registries for oncological trials. The automatic release of the CSRs might have led to better reporting of harms in other sources of data, but this does not seem to be the case. Additionally, we systematically examined predefined variables in a relatively large sample of trials.

Our study has some limitations. First, we focused on oncology trials, and our findings might not be applicable to other fields of medicine. However, our results, together with results from previous studies, suggest that the reporting of harms is better in CSRs than trial registries and journal publications across all specialities. Second, we examined only two clinical trial registries, and more information might be available from other registries; however, ClinicalTrials.gov and EUCTR are two of the most-used registries, and information available elsewhere is unlikely to substantially alter our conclusions.

Our study has important implications for both research and practice. Our results suggest that any systematic review or other assessment of harms associated with oncological treatments would have to rely on CSRs for making the soundest conclusions. If such an assessment relies on data from only publications and trial registries, it will only be able to include a subset of the available data. This is problematic for several reasons, namely reduced power to detect differences between groups and the fact that the data reported in registries and publications might vary systematically from data not reported. Additionally, we have shown marked discrepancies in data reported in CSRs and other sources, especially for withdrawals due to adverse events; therefore, we consider results based on CSRs more reliable. However, while we believe that CSRs are currently the most reliable source of data on harms, using CSRs might currently not be feasible. First, even though CSRs contained more data than other sources, important information was still missing in a significant number of CSRs. Secondly, we identified a substantial delay between the completion of trials and the availability of a CSR. To solve the problem of missing data in CSRs, we suggest that regulatory authorities make stricter requirements to the quality of submitted CSRs. The issue of delay in access is complicated by the fact that the EMA does not release CSRs until the procedure for which they were submitted is completed; however, we identified substantial delay from completion of the procedure to availability of CSR. By decreasing this delay to the absolute minimum, the overall delay could be reduced substantially. Currently, the EMA are not releasing any CSRs due to the agency’s move to Amsterdam, and in a reply to an open letter from IQWIQ and Cochrane the agency would not commit to reinitiating publication of CSRs [119].

In addition, improving reporting of harms in journal publications would be valuable, as this is a very accessible source of information and as publications are often available earlier than CSRs. By actively enforcing the CONSORT statement and the extension for harms, journals could help improve the reporting of harms. Also, the CONSORT extension for harms, which was released in 2004, could be updated to better reflect new opportunities for data-sharing and new knowledge on reporting of harms.

In addition, current estimates of the harms of oncological treatments based on published data might not be accurate and not able to inform clinical practice. Because oncological treatments are generally toxic, the harms profile is an important piece of information for assessing the benefit/harm balance, and true informed consent is only possible if the estimate of harms is accurate.

We suggest that future studies comparing reporting of harms in different sources should focus on more detailed aspects of harms reporting, e.g. whether information on adverse events by System Organ Class and Preferred Term levels are available.

## Conclusions

Harms in trials evaluating targeted therapy and immunotherapy for cancer are reported in more detail and more reliably in CSRs than in trial registries and journal publications. This finding confirms previous results and suggests that CSR would provide more complete data on harms of oncological treatments and ideally systematic reviewers should have access to CSRs for providing accurate evidence synthesis. Thus, we consider it vital that regulators start routinely releasing CSRs.

## Supplementary Information

**Additional file 1. **Characteristics of included trials.

## Data Availability

The CSRs used for this study are available at the EMA’s clinical data Website. All data files and the code for the statistical analysis are available from the Open Science Framework database (link: https://osf.io/mabyu/?view_only=d58aea7810234c229e593549569bc42d).

## Abbreviations

ATC: Anatomic therapeutic chemical
CSR: Clinical study report
CTCAE: Common Terminology Criteria for Adverse Events
EMA: European Medicines Agency
EUCTR: European Clinical Trials Register
RCT: Randomised controlled trial

## References

[1] Sackett DL, Rosenberg WMC, Gray JAM, Haynes RB, Richardson WS (1996). Evidence based medicine: what it is and what it isn’t. BMJ..

[2] Dickersin K (1990). The existence of publication bias and risk factors for its occurrence. JAMA..

[3] Dwan K, Gamble C, Williamson PR, Kirkham JJ, Reporting Bias Group (2013). Systematic review of the empirical evidence of study publication bias and outcome reporting bias - an updated review. PloS One.

[4] Scherer RW, Meerpohl JJ, Pfeifer N, Schmucker C, Schwarzer G, von Elm E (2018). Full publication of results initially presented in abstracts. Cochrane Database Syst Rev.

[5] Hodkinson A, Kirkham JJ, Tudur-Smith C, Gamble C. Reporting of harms data in RCTs: a systematic review of empirical assessments against the CONSORT harms extension. BMJ Open. 2013;3. 10.1136/bmjopen-2013-003436. 10.1136/bmjopen-2013-003436 PMC3787508 24078752

[6] The International Council for Harmonisation of Technical Requirements for Human Use (ICH) (1996). Guideline E3 - Structure and Content of Clinical Study Reports.

[7] Gøtzsche PC, Jørgensen AW. Opening up data at the European medicines agency. BMJ. 2011;342. 10.1136/bmj.d2686. 10.1136/bmj.d2686 21558364

[8] European Medicines Agency. Clinical data publication (2018). European Medicines Agency.

[9] Maund E, Guski LS, Gøtzsche PC (2017). Considering benefits and harms of duloxetine for treatment of stress urinary incontinence: a meta-analysis of clinical study reports. CMAJ.

[10] Jefferson T, Jones M, Doshi P, Spencer EA, Onakpoya I, Heneghan CJ (2014). Oseltamivir for influenza in adults and children: systematic review of clinical study reports and summary of regulatory comments. BMJ..

[11] Rohner E, Grabik M, Tonia T, Jüni P, Pétavy F, Pignatti F (2017). Does access to clinical study reports from the European medicines agency reduce reporting biases? A systematic review and meta-analysis of randomized controlled trials on the effect of erythropoiesis-stimulating agents in cancer patients. Plos One.

[12] Sharma T, Guski LS, Freund N, Gøtzsche PC (2016). Suicidality and aggression during antidepressant treatment: systematic review and meta-analyses based on clinical study reports. BMJ..

[13] Hodkinson A, Dietz KC, Lefebvre C, Golder S, Jones M, Doshi P (2018). The use of clinical study reports to enhance the quality of systematic reviews: a survey of systematic review authors. Syst Rev.

[14] Golder S, Loke YK, Wright K, Sterrantino C (2016). Most systematic reviews of adverse effects did not include unpublished data. J Clin Epidemiol.

[15] Wieseler B, Kerekes MF, Vervoelgyi V, McGauran N, Kaiser T. Impact of document type on reporting quality of clinical drug trials: a comparison of registry reports, clinical study reports, and journal publications. BMJ. 2012;344. 10.1136/bmj.d8141. 10.1136/bmj.d8141 22214759

[16] Wieseler B, Wolfram N, McGauran N, Kerekes MF, Vervölgyi V, Kohlepp P (2013). Completeness of reporting of patient-relevant clinical trial outcomes: comparison of unpublished clinical study reports with publicly available data. Plos Med.

[17] Maund E, Tendal B, Hróbjartsson A, Jørgensen KJ, Lundh A, Schroll J (2014). Benefits and harms in clinical trials of duloxetine for treatment of major depressive disorder: comparison of clinical study reports, trial registries, and publications. BMJ..

[18] Hodkinson A, Gamble C, Smith CT (2016). Reporting of harms outcomes: a comparison of journal publications with unpublished clinical study reports of orlistat trials. Trials..

[19] Schroll JB, Penninga EI, Gøtzsche PC (2016). Assessment of adverse events in protocols, clinical study reports, and published papers of trials of orlistat: a document analysis. Plos Med.

[20] Mayo-Wilson E, Fusco N, Hong H, Li T, Canner JK, Dickersin K (2019). Opportunities for selective reporting of harms in randomized clinical trials: selection criteria for non-systematic adverse events. Trials..

[21] European Medicines Agency (2019). European Medicines Agency policy on publication of clinical data for medicinal products for human use.

[22] European Commission. Union register of medicinal products. https://ec.europa.eu/health/documents/community-register/html/index_en.htm#foot2. Accessed 3 Feb 2020.

[23] Barlesi F, Garon EB, Kim D-W, Felip E, Han J-Y, Kim J-H, Ahn M-J, Fidler MJ, Gubens MA, de Castro G, Surmont V, Li Q, Deitz AC, Lubiniecki GM, Herbst RS. Health-related quality of life in KEYNOTE-010: a phase II/III study of pembrolizumab versus docetaxel in patients with previously treated advanced, programmed death ligand 1–expressing NSCLC. J Thor Oncol. 2019;14(5):793–801. 10.1016/j.jtho.2019.01.016 30711649

[24] Basch EM, Scholz M, de Bono JS, Vogelzang N, de Souza P, Marx G, Vaishampayan U, George S, Schwarz JK, Antonarakis ES, O'Sullivan JM, Kalebasty AR, Chi KN, Dreicer R, Hutson TE, Dueck AC, Bennett AV, Dayan E, Mangeshkar M, Holland J, Weitzman AL, Scher HI. Cabozantinib versus mitoxantrone-prednisone in symptomatic metastatic castration-resistant prostate cancer: a randomized phase 3 trial with a primary pain endpoint. Eur Urol. 2019;75(6):929–37. 10.1016/j.eururo.2018.11.033 PMC6876845 30528222

[25] Batchelor TT, Mulholland P, Neyns N, Nabors LB, Campone M, Wick A, Mason W, Mikkelsen T, Phuphanich S, Ashby LS, DeGroot J, Gattamaneni R, Cher L, Rosenthal M, Payer F, Jürgensmeier JM, Jain RK, Sorensen AG, Xu J, Liu Q, van den Bent M. Phase III randomized trial comparing the efficacy of cediranib as monotherapy, and in combination with lomustine, versus lomustine alone in patients with recurrent glioblastoma. J Clin Oncol. 2013;31(26):3212–8. 10.1200/JCO.2012.47.2464 PMC4021043 23940216

[26] Bhadhuri A, Insinga R, Guggisberg P, Panje C, Schwenkglenks M. Cost effectiveness of pembrolizumab vs chemotherapy as firstline treatment for metastatic NSCLC that expresses high levels of PD-L1 in Switzerland. Swiss Med Wkly. 2019;149:w20170. 10.4414/smw.2019.20170 31880807

[27] Borghaei H, Paz-Ares L, Horn L, Spigel DR, Steins M, Ready NE, Chow LQ, Vokes EE, Felip E, Holgado E, Barlesi F, Kohlhäufl M, Arrieta O, Burgio MA, Fayette J, Lena H, Poddubskaya E, Gerber DE, Gettinger SN, Rudin CM, Rizvi N, Crinò L, Blumenschein GR, Antonia SJ, Dorange C, Harbison CT, Finckenstein FG, Brahmer JR. Nivolumab versus Docetaxel in Advanced Nonsquamous Non–Small-Cell Lung Cancer. New Engl J Med. 2015;373(17):1627–39. 10.1056/NEJMoa1507643 PMC5705936 26412456

[28] Brahmer JR, Rodríguez-Abreu D, Robinson AG, Hui R, Csőszi T, Fülöp A, Gottfried M, Peled N, Tafreshi A, Cuffe S, O'Brien M, Rao S, Hotta K, Zhang J, Lubiniecki GM, Deitz AC, Rangwala R, Reck M. Health-related quality-of-life results for pembrolizumab versus chemotherapy in advanced, PD-L1-positive NSCLC (KEYNOTE-024): a multicentre, international, randomised, open-label phase 3 trial. Lancet Oncol. 2017;18(12):1600–9. 10.1016/S1470-2045(17)30690-3 29129441

[29] Buzzoni R, Carnaghi C, Strosberg J, Fazio N, Singh S, Herbst F, Ridolfi A, Pavel M, Wolin E, Valle J, Oh D-Y, Yao J, Pommier R. Impact of prior therapies on everolimus activity: an exploratory analysis of RADIANT-4. OncoTargets Ther. 2017;10:5013–30. 10.2147/OTT.S142087 PMC5652899 29081664

[30] Cappuzzo F, Ciuleanu T, Stelmakh L, Cicenas S, Szczésna A, Juhász E, Esteban E, Molinier O, Brugger W, Melezínek I, Klingelschmitt G, Klughammer B, Giaccone G. Erlotinib as maintenance treatment in advanced non-small-cell lung cancer: a multicentre, randomised, placebo-controlled phase 3 study. Lancet Oncol. 2010;11(6):521–9. 10.1016/S1470-2045(10)70112-1 20493771

[31] Carlino MS, Long GV, Schadendorf D, Robert C, Ribas A, Richtig E, Nyakas M, Caglevic C, Tarhini A, Blank C, Hoeller C, Bar-Sela G, Barrow C, Wolter P, Zhou H, Emancipator K, Jensen EH, Ebbinghaus S, Ibrahim N, Daud A, Outcomes by line of therapy and programmed death ligand 1 expression in patients with advanced melanoma treated with pembrolizumab or ipilimumab in KEYNOTE-006: a randomised clinical trial. Eur J Cancer. 2018;101:236–43. 10.1016/j.ejca.2018.06.034 30096704

[32] Cella D, Escudier B, Tannir NM, Powles T, Donskov F, Peltola K, Schmidinger M, Heng DYC, Mainwaring PN, Hammers HJ, Lee JL, Roth BJ, Marteau F, Williams P, Baer J, Mangeshkar M, Scheffold C, Hutson TE, Pal S, Motzer RJ, Choueiri TK. Quality of life outcomes for cabozantinib versus everolimus in patients with metastatic renal cell carcinoma: METEOR phase III randomized trial. J Clin Oncol. 2018;36(8):757–64. 10.1200/JCO.2017.75.2170 PMC6804841 29377755

[33] Cella D, Grünwald V, Nathan P, Doan J, Dastani H, Taylor F, Bennett B, DeRosa M, Berry S, Broglio K, Berghorn E, Motzer RJ. Quality of life in patients with advanced renal cell carcinoma given nivolumab versus everolimus in CheckMate 025: a randomised, open-label, phase 3 trial. Lancet Oncol. 2016;17(7):994–1003. 10.1016/S1470-2045(16)30125-5 PMC5521044 27283863

[34] Choueiri TK, Escudier B, Powles T, Tannir NM, Mainwaring PN, Rini BI, Hammers HJ, Donskov F, Roth BJ, Peltola K, Lee JL, Heng DYC, Schmidinger M, Agarwal N, Sternberg CN, McDermott DF, Aftab DT, Hessel C, Scheffold C, Schwab G, Hutson TE, Pal S, Motzer RJ, Cabozantinib versus everolimus in advanced renal cell carcinoma (METEOR): final results from a randomised, open-label, phase 3 trial. Lancet Oncol. 2016;17(7):917–27. 10.1016/S1470-2045(16)30107-3 27279544

[35] Choueiri TK, Escudier B, Powles T, Mainwaring PN, Rini BI, Donskov F, Hammers H, Hutson TE, Lee J-L, Peltola K, Roth BJ, Bjarnason GA, Géczi L, Keam B, Maroto P, Heng DYC, Schmidinger M, Kantoff PW, Borgman-Hagey A, Hessel C, Scheffold C, Schwab GM, Tannir NM, Motzer RJ. Cabozantinib versus everolimus in advanced renal-cell carcinoma. N Engl J Med. 2015;373(19):1814–23. 10.1056/NEJMoa1510016 PMC5024539 26406150

[36] Ciuleanu T, Tsai C-M, Tsao C-J, Milanowski J, Amoroso D, Heo DS, Groen HJM, Szczesna A, Chung C-Y, Chao T-Y, Middleton G, Zeaiter A, Klingelschmitt G, Klughammer B, Thatcher N. A phase II study of erlotinib in combination with bevacizumab versus chemotherapy plus bevacizumab in the first-line treatment of advanced non-squamous non-small cell lung cancer. Lung Cancer. 2013;82(2):276–81. 10.1016/j.lungcan.2013.08.002 23992877

[37] Clement PM, Gauler T, Machiels JP, Haddad RI, Fayette J, Licitra LF, Tahara M, Cohen EEW, Cupissol D, Grau JJ, Guigay J, Caponigro F, de Castro G, de Souza Viana L, Keilholz U, del Campo JM, Cong XJ, Ehrnrooth E, Vermorken JB. Afatinib versus methotrexate in older patients with secondline recurrent and/or metastatic head and neck squamous cell carcinoma: subgroup analysis of the LUX-Head & Neck 1 trial. Ann Oncol. 2016;27(8):1585–93. 10.1093/annonc/mdw151 PMC4959921 27084954

[38] Cohen EEW, Licitra LF, Burtness B, Fayette J, Gauler T, Clement PM, Grau JJ, del Campo JM, Mailliez A, Haddad RI, Vermorken JB, Tahara M, Guigay J, Geoffrois L, Merlano MC, Dupuis N, Krämer N, Cong XJ, Gibson N, Solca F, Ehrnrooth E, Machiels J-PH. Biomarkers predict enhanced clinical outcomes with afatinib versus methotrexate in patients with second-line recurrent and/or metastatic head and neck cancer. Ann Oncol. 2017;28 (10):2526–32. 10.1093/annonc/mdx344 PMC5834024 28961833

[39] Costa C, Molina MA, Drozdowskyj A, Giménez-Capitán A, Bertran-Alamillo J, Karachaliou N, Gervais R, Massuti B, Wei J, Moran T, Majem M, Felip E, Carcereny E, Garcia-Campelo R, Viteri S, Taron M, Ono M, Giannikopoulos P, Bivona T, Rosell R. The impact of T790M Mutations and mRNA expression on outcome in patients with -mutant NSCLC treated with erlotinib or chemotherapy in the randomized phase III EURTAC trial . Clin Cancer Res. 2014;20(7):2001–10. 10.1158/1078-0432.CCR-13-2233 24493829

[40] Cristofanilli M, DeMichele A, Giorgetti C, Turner NC, Slamon DJ, Im S-A, Masuda N, Verma S, Loi S, Colleoni M, Theall KP, Huang X, Liu Y, Bartlett CH. Predictors of prolonged benefit from palbociclib plus fulvestrant in women with endocrine-resistant hormone receptor–positive/human epidermal growth factor receptor 2–negative metastatic breast cancer in PALOMA-3. Eur J Cancer. 2018;104:21–31. 10.1016/j.ejca.2018.08.011 30308388

[41] Dueck AC, Scher HI, Bennett AV, Mazza GL, Thanarajasingam G, Schwab G, Weitzman AL, Rogak LJ, Basch E. Assessment of adverse events from the patient perspective in a phase 3 metastatic castration-resistant prostate cancer clinical trial. JAMA Oncol. 2020;6(2):e193332. 10.1001/jamaoncol.2019.3332 PMC6764147 31556911

[42] Elisei R, Schlumberger MJ, Müller SP, Schöffski P, Brose MS, Shah MH, Licitra L, Jarzab B, Medvedev V, Kreissl MC, Niederle B, Cohen EEW, Wirth LJ, Ali H, Hessel C, Yaron Y, Ball D, Nelkin B, Sherman SI. Cabozantinib in progressive medullary thyroid cancer. J Clin Oncol. 2013;31(29):3639–46. 10.1200/JCO.2012.48.4659 PMC4164813 24002501

[43] Escudier B, Motzer RJ, Sharma P, Wagstaff J, Plimack ER, Hammers HJ, Donskov F, Gurney H, Sosman JA, Zalewski PG, Harmenberg U, McDermott DF, Choueiri TK, Richardet M, Tomita Y, Ravaud A, Doan J, Zhao H, Hardy H, George S. Treatment beyond progression in patients with advanced renal cell carcinoma treated with nivolumab in CheckMate 025. Eur Urol. 2017;72(3):368–76. 10.1016/j.eururo.2017.03.037 28410865

[44] Escudier B, Powles T, Motzer RJ, Olencki T, Frontera oA, Oudard S, Rolland F, Tomczak P, Castellano D, Appleman LJ, Drabkin H, Vaena D, Milwee S, Youkstetter J, Lougheed JC, Bracarda S, Choueiri TK. Cabozantinib, a new standard of care for patients with advanced renal cell carcinoma and bone metastases? Subgroup analysis of the METEOR Trial. J Clin Oncol. 2018;36(8):765–72. 10.1200/JCO.2017.74.7352 PMC6804840 29309249

[45] Escudier B, Sharma P, McDermott DF, George S, Hammers HJ, Srinivas S, Tykodi SS, Sosman JA, Procopio G, Plimack ER, Castellano D, Gurney H, Donskov F, Peltola K, Wagstaff J, Gauler TC, Ueda T, Zhao H, Waxman IM, Motzer RJ. CheckMate 025 randomized phase 3 study: outcomes by key baseline factors and prior therapy for nivolumab versus everolimus in advanced renal cell carcinoma. Eur Urol. 2017;72(6):962–71. 10.1016/j.eururo.2017.02.010 28262413

[46] Evans TRJ, Kudo M, Finn RS, Han K-H, Cheng A-L, Ikeda M, Kraljevic S, Ren M, Dutcus CE, Piscaglia F, Sung MW. Urine protein:creatinine ratio vs 24-hour urine protein for proteinuria management: analysis from the phase 3 REFLECT study of lenvatinib vs sorafenib in hepatocellular carcinoma. Br J Cancer. 2019;121(3):218–21. 10.1038/s41416-019-0506-6 PMC6738107 31249394

[47] Fazio N, Buzzoni R, Fave GD, Tesselaar ME, Wolin E, Van Cutsem E, Tomassetti P, Strosberg J, Voi M, Bubuteishvili-Pacaud L, Ridolfi A, Herbst F, Tomasek J, Singh S, Pavel M, Kulke MH, Valle JW, Yao JC. Everolimus in advanced, progressive, well-differentiated, non-functional neuroendocrine tumors: RADIANT-4 lung subgroup analysis. Cancer Sci. 2018;109(1):174–81. 10.1111/cas.13427 PMC5765303 29055056

[48] Finn RS, Martin M, Rugo HS, Jones S, Im S-A, Gelmon K, Harbeck N, Lipatov ON, Walshe JM, Moulder S, Gauthier E, Lu DR, Randolph S, Diéras V, Slamon DJ. Palbociclib and letrozole in advanced breast cancer. N Engl J Med. 2016;375(20):1925–36. 10.1056/NEJMoa1607303 27959613

[49] Gadgeel S, Goss G, Soria J-C, Felip E, Georgoulias V, Lu S, Cobo M, Syrigos K, Lee KH, Göker E, Guclu SZ, Isla D, Morabito A, Dupuis N, Bühnemann C, Krämer N, Solca F, Ehrnrooth E, Ardizzoni A. Evaluation of the VeriStrat® serum protein test in patients with advanced squamous cell carcinoma of the lung treated with second-line afatinib or erlotinib in the phase III LUX-Lung 8 study. Lung Cancer. 2017;109:101–8. 10.1016/j.lungcan.2017.05.010 28577938

[50] Goss GD, Arnold A, Shepherd FA, Dediu M, Ciuleanu T-E, Fenton D, Zukin M, Walde D, Laberge F, Vincent MD, Ellis PM, Laurie SA, Ding K, Frymire E, Gauthier I, Leighl NB, Ho C, Noble J, Lee CW, Seymour L. Randomized, double-blind trial of carboplatin and paclitaxel with either daily oral cediranib or placebo in advanced non–small-cell lung cancer: NCIC Clinical Trials Group BR24 Study. J Clin Oncol. 2010;28(1):49–55. 10.1200/JCO.2009.22.9427 19917841

[51] Goss GD, Felip E, Cobo M, Lu S, Syrigos K, Lee KH, Göker E, Georgoulias V, Li W, Guclu S, Isla D, Min YJ, Morabito A, Ardizzoni A, Gadgeel SM, Fülöp A, Bühnemann C, Gibson N, Krämer N, Solca F, Cseh A, Ehrnrooth E, Soria J-C. Association of mutations with clinical outcomes of afatinib- or erlotinib-treated patients with lung squamous cell carcinoma . JAMA Oncol. 2018;4(9):1189. 10.1001/jamaoncol.2018.0775 PMC6143014 29902295

[52] Harbeck N, Iyer S, Turner N, Cristofanilli M, Ro J, André F, Loi S, Verma S, Iwata H, Bhattacharyya H, Puyana Theall K, Bartlett CH, Loibl S. Quality of life with palbociclib plus fulvestrant in previously treated hormone receptor-positive, HER2-negative metastatic breast cancer: patient-reported outcomes from the PALOMA-3 trial. Ann Oncol. 2016;27(6):1047–54. 10.1093/annonc/mdw139 PMC4880065 27029704

[53] Herbst RS, Baas P, Perez-Gracia JL, Felip E, Kim D-W, Han J-Y, Molina JR, Kim J-H, Dubos Arvis C, Ahn M-J, Majem M, Fidler MJ, Surmont V, de Castro G, Garrido M, Shentu Y, Emancipator K, Samkari A, Jensen EH, Lubiniecki GM, Garon EB. Use of archival versus newly collected tumor samples for assessing PD-L1 expression and overall survival: an updated analysis of KEYNOTE-010 trial. Ann Oncol. 2019;30(2):281–9. 10.1093/annonc/mdy545 PMC6931268 30657853

[54] Herbst RS, Ansari R, Bustin F, Flynn P, Hart L, Otterson GA, Vlahovic G, Soh C-H, O'Connor P, Hainsworth J. Efficacy of bevacizumab plus erlotinib versus erlotinib alone in advanced non-small-cell lung cancer after failure of standard first-line chemotherapy (BeTa): a double-blind, placebo-controlled, phase 3 trial. Lancet. 2011;377(9780):1846–54. 10.1016/S0140-6736(11)60545-X PMC4134127 21621716

[55] Herbst RS, Baas P, Kim D-W, Felip E, Pérez-Gracia JL, Han J-Y, Molina J, Kim J-H, Arvis CD, Ahn M-J, Majem M, Fidler MJ, de Castro G, Garrido M, Lubiniecki GM, Shentu Y, Im E, Dolled-Filhart M, Garon EB. Pembrolizumab versus docetaxel for previously treated, PD-L1-positive, advanced non-small-cell lung cancer (KEYNOTE-010): a randomised controlled trial. Lancet. 2016;387(10027):1540–50. 10.1016/S0140-6736(15)01281-7 26712084

[56] Hodi FS, Chesney J, Pavlick AC, Robert C, Grossmann KF, McDermott DF, Linette GP, Meyer N, Giguere JK, Agarwala SS, Shaheen M, Ernstoff MS, Minor DR, Salama AK, Taylor MH, Ott PA, Horak C, Gagnier P, Jiang J, Wolchok JD, Postow MA. Combined nivolumab and ipilimumab versus ipilimumab alone in patients with advanced melanoma: 2-year overall survival outcomes in a multicentre, randomised, controlled, phase 2 trial. Lancet Oncol. 2016;17(11):1558–68. 10.1016/S1470-2045(16)30366-7 PMC5630525 27622997

[57] Hodi FS, Chiarion-Sileni V, Gonzalez R, Grob J-J, Rutkowski P, Cowey CL, Lao CD, Schadendorf D, Wagstaff J, Dummer R, Ferrucci PF, Smylie M, Hill A, Hogg D, Marquez-Rodas I, Jiang J, Rizzo J, Larkin J, Wolchok JD. Nivolumab plus ipilimumab or nivolumab alone versus ipilimumab alone in advanced melanoma (CheckMate 067): 4-year outcomes of a multicentre, randomised, phase 3 trial. Lancet Oncol. 2018;19(11):1480–92. 10.1016/S1470-2045(18)30700-9 30361170

[58] Karachaliou N, Gimenez-Capitan A, Drozdowskyj A, Viteri S, Moran T, Carcereny E, Massuti B, Vergnenegre A, de Marinis F, Molina MA, Teixido C, Rosell R. ROR1 as a novel therapeutic target for EGFR-mutant non-small-cell lung cancer patients with the EGFR T790M mutation. Transl Lung Cancer Res. 2014;3(3). 10.3978/j.issn.2218-6751.2014.03.02 PMC4367691 25806291

[59] Karachaliou N, Mayo-de las Casas C, Queralt C, de Aguirre I, Melloni B, Cardenal F, Garcia-Gomez R, Massuti B, Sánchez JM, Porta R, Ponce-Aix S, Moran T, Carcereny E, Felip E, Bover I, Insa A, Reguart N, Isla D, Vergnenegre A, de Marinis F, Gervais R, Corre R, Paz-Ares L, Morales-Espinosa D, Viteri S, Drozdowskyj A, Jordana-Ariza N, Ramirez-Serrano JL, Molina-Vila MA, Rosell R. Association of L858R Mutation in Circulating Free DNA With Survival in the EURTAC Trial . JAMA Oncol. 2015;1(2):149. 10.1001/jamaoncol.2014.257 26181014

[60] Kiyota N, Schlumberger M, Muro K, Ando Y, Takahashi S, Kawai Y, Wirth L, Robinson B, Sherman S, Suzuki T, Fujino K, Gupta A, Hayato S, Tahara M. Subgroup analysis of Japanese patients in a phase 3 study of lenvatinib in radioiodinerefractory differentiated thyroid cancer. Cancer Sci. 2015;106(12):1714–21. 10.1111/cas.12826 PMC4714672 26426092

[61] Kudo M, Finn RS, Qin S, Han K-H, Ikeda K, Piscaglia F, Baron A, Park J-W, Han G, Jassem J, Blanc JF, Vogel A, Komov D, Evans TRJ, Lopez C, Dutcus C, Guo M, Saito K, Kraljevic S, Tamai T, Ren M, Cheng A-L. Lenvatinib versus sorafenib in first-line treatment of patients with unresectable hepatocellular carcinoma: a randomised phase 3 noninferiority trial. Lancet. 2018;391(10126):1163–73. 10.1016/S0140-6736(18)30207-1 29433850

[62] Larkin J, Chiarion-Sileni V, Gonzalez R, Grob JJ, Cowey CL, Lao CD, Schadendorf D, Dummer R, Smylie M, Rutkowski P, Ferrucci PF, Hill A, Wagstaff J, Carlino MS, Haanen JB, Maio M, Marquez-Rodas I, McArthur GA, Ascierto PA, Long GV, Callahan MK, Postow MA, Grossmann K, Sznol M, Dreno B, Bastholt L, Yang A, Rollin LM, Horak C, Hodi FS, Wolchok JD. Combined Nivolumab and Ipilimumab or Monotherapy in Untreated Melanoma. N Engl J Med. 2015;373(1):23–34. 10.1056/NEJMoa1504030 PMC5698905 26027431

[63] Larkin J, Chiarion-Sileni V, Gonzalez R, Grob J-J, Rutkowski P, Lao CD, Cowey CL, Schadendorf D, Wagstaff J, Dummer R, Ferrucci PF, Smylie M, Hogg D, Hill A, Márquez-Rodas I, Haanen J, Guidoboni M, Maio M, Schöffski P, Carlino MS, Lebbé C, McArthur G, Ascierto PA, Daniels GA, Long GV, Bastholt L, Rizzo JI, Balogh A, Moshyk A, Hodi FS, Wolchok JD. Five-year survival with combined nivolumab and ipilimumab in advanced melanoma. N Engl J Med. 2019;381(16):1535–46. 10.1056/NEJMoa1910836 31562797

[64] Laurie SA, Solomon BJ, Seymour L, Ellis PM, Goss GD, Shepherd FA, Boyer MJ, Arnold AM, Clingan P, Laberge F, Fenton D, Hirsh V, Zukin M, Stockler MR, Lee CW, Chen EX, Montenegro A, Ding K, Bradbury PA. Randomised, double-blind trial of carboplatin and paclitaxel with daily oral cediranib or placebo in patients with advanced non-small cell lung cancer: NCIC Clinical Trials Group study BR29. Eur J Cancer. 2014;50(4):706–12. 10.1016/j.ejca.2013.11.032 24360368

[65] Ledermann JA, Embleton AC, Raja F, Perren TJ, Jayson GC, Rustin GJS, Kaye SB, Hirte H, Eisenhauer E, Vaughan M, Friedlander M, González-Martín A, Stark D, Clark E, Farrelly L, Swart AM, Cook A, Kaplan RS, Parmar MKB. Cediranib in patients with relapsed platinum-sensitive ovarian cancer (ICON6): a randomised, double-blind, placebo controlled phase 3 trial. Lancet. 2016;387(10023):1066–74. 10.1016/S0140-6736(15)01167-8 27025186

[66] Loibl S, Turner NC, Ro J, Cristofanilli M, Iwata H, Im S-A, Masuda N, Loi S, André F, Harbeck N, Verma S, Folkerd E, Theall KP, Hoffman J, Zhang K, Bartlett CH, Dowsett M. Palbociclib combined with fulvestrant in premenopausal women with advanced breast cncer and prior progression on endocrine therapy: PALOMA‐3 results. Oncologist. 2017;22(9):1028–38. 10.1634/theoncologist.2017-0072 PMC5599195 28652278

[67] Lu S, Li W, Zhou C, Hu C-P, Qin S, Cheng G, Feng J, Wang J, Cseh A, Peil B, Gibson N, Ehrnrooth E, Zhang L. Afatinib vs erlotinib for second-line treatment of Chinese patients with advanced squamous cell carcinoma of the lung. OncoTargets Ther. 2018;11:8565–73. 10.2147/OTT.S161506 PMC6292388 30573970

[68] Machiels J-PH, Haddad RI, Fayette J, Licitra LF, Tahara M, Vermorken JB, Clement PM, Gauler T, Cupissol D, Grau JJ, Guigay J, Caponigro F, de Castro G, de Souza Viana L, Keilholz U, del Campo JM, Cong XJ, Ehrnrooth E, Cohen EEW. Afatinib versus methotrexate as second-line treatment in patients with recurrent or metastatic squamous-cell carcinoma of the head and neck progressing on or after platinum-based therapy (LUX-Head & Neck 1): an open-label, randomised phase 3 trial. Lancet Oncol. 2015;16(5):583–94. 10.1016/S1470-2045(15)70124-5 25892145

[69] Masuda N, Inoue K, Nakamura R, Rai Y, Mukai H, Ohno S, Hara F, Mori Y, Hashigaki S, Muramatsu Y, Nagasawa T, Umeyama Y, Huang X, Iwata H. Palbociclib in combination with fulvestrant in patients with hormone receptorpositive, human epidermal growth factor receptor 2-negative advanced breast cancer: PALOMA-3 subgroup analysis of Japanese patients. Int J Clin Oncol. 2019;24(3):262–73. 10.1007/s10147-018-1359-3 PMC6399170 30392115

[70] McGuire WP, Penson RT, Gore M, Herraez AC, Peterson P, Shahir A, Ilaria R. Randomized phase II study of the PDGFRα antibody olaratumab plus liposomal doxorubicin versus liposomal doxorubicin alone in patients with platinum-refractory or platinum-resistant advanced ovarian cancer. BMC Cancer. 2018;18(1). 10.1186/s12885-018-5198-4 PMC6307114 30591028

[71] Miller VA, Hirsh V, Cadranel J, Chen Y-M, Park K, Kim S-W, Zhou C, Su W-C, Wang M, Sun Y, Heo DS, Crino L, Tan E-H, Chao T-Y, Shahidi M, Cong XJ, Lorence RM, Yang JC-H. Afatinib versus placebo for patients with advanced, metastatic non-small-cell lung cancer after failure of erlotinib, gefitinib, or both, and one or two lines of chemotherapy (LUX-Lung 1): a phase 2b/3 randomised trial. Lancet Oncol. 2012;13(5):528–38. 10.1016/S1470-2045(12)70087-6 22452896

[72] Motzer RJ, Escudier B, McDermott DF, George S, Hammers HJ, Srinivas S, Tykodi SS, Sosman JA, Procopio G, Plimack ER, Castellano D, Choueiri TK, Gurney H, Donskov F, Bono P, Wagstaff J, Gauler TC, Ueda T, Tomita Y, Schutz FA, Kollmannsberger C, Larkin J, Ravaud A, Simon JS, Xu L-A, Waxman IM, Sharma P. Nivolumab versus everolimus in avanced renal-cell carcinoma. N Engl J Med. 2015;373(19):1803–13. 10.1056/NEJMoa1510665 PMC5719487 26406148

[73] Mukai H, Shimizu C, Masuda N, Ohtani S, Ohno S, Takahashi M, Yamamoto Y, Nishimura R, Sato N, Ohsumi S, Iwata H, Mori Y, Hashigaki S, Muramatsu Y, Nagasawa T, Umeyama Y, Lu DR, Toi M. Palbociclib in combination with letrozole in patients with estrogen receptor–positive, human epidermal growth factor receptor 2–negative advanced breast cancer: PALOMA-2 subgroup analysis of Japanese patients. Int J Clin Oncol. 2019;24(3):274–87. 10.1007/s10147-018-1353-9 PMC6399183 30515674

[74] Mulders P, Hawkins R, Nathan P, de Jong I, Osanto S, Porfiri E, Protheroe A, van Herpen CML, Mookerjee B, Pike L, Jürgensmeier JM, Gore ME. Cediranib monotherapy in patients with advanced renal cell carcinoma: results of a randomised phase II study. Eur J Cancer. 2012;48(4):527–37. 10.1016/j.ejca.2011.12.022 22285180

[75] Pavel ME, Singh S, Strosberg JR, Bubuteishvili-Pacaud L, Degtyarev E, Neary MP, Carnaghi C, Tomasek J, Wolin E, Raderer M, Lahner H, Valle JW, Pommier R, Van Cutsem E, Tesselaar MET, Fave GD, Buzzoni R, Hunger M, Eriksson J, Cella D, Ricci J-F, Fazio N, Kulke MH, Yao JC. Health-related quality of life for everolimus versus placebo in patients with advanced, non-functional, well-differentiated gastrointestinal or lung neuroendocrine tumours (RADIANT-4): a multicentre, randomised, double-blind, placebo-controlled, phase 3 trial. Lancet Oncol. 2017;18 (10):1411–22. 10.1016/S1470-2045(17)30471-0 28838862

[76] Petrella TM, Robert C, Richtig E, Miller WH, Masucci GV, Walpole E, Lebbe C, Steven N, Middleton MR, Hille D, Zhou W, Ibrahim N, Cebon J. Patient-reported outcomes in KEYNOTE-006, a randomised study of pembrolizumab versus ipilimumab in patients with advanced melanoma. Eur J Cancer. 2017;86:115–24. 10.1016/j.ejca.2017.08.032 28987768

[77] Postow MA, Chesney J, Pavlick AC, Robert C, Grossmann K, McDermott D, Linette GP, Meyer N, Giguere JK, Agarwala SS, Shaheen M, Ernstoff MS, Minor D, Salama AK, Taylor M, Ott PA, Rollin LM, Horak C, Gagnier P, Wolchok JD, Hodi FS. Nivolumab and Ipilimumab versus Ipilimumab in Untreated Melanoma. N Engl J Med. 2015;372(21):2006–17. 10.1056/NEJMoa1414428 PMC5744258 25891304

[78] Reck M, Brahmer J, Bennett B, Taylor F, Penrod JR, DeRosa M, Dastani H, Spigel DR, Gralla RJ. Evaluation of health-related quality of life and symptoms in patients with advanced non-squamous non-small cell lung cancer treated with nivolumab or docetaxel in CheckMate 057. Eur J Cancer. 2018;102:23–30 10.1016/j.ejca.2018.05.005 30103096

[79] Reck M, Rodríguez-Abreu D, Robinson AG, Hui R, Csőszi T, Fülöp A, Gottfried M, Peled N, Tafreshi A, Cuffe S, O’Brien M, Rao S, Hotta K, Leiby MA, Lubiniecki GM, Shentu Y, Rangwala R, Brahmer JR. Pembrolizumab versus chemotherapy for PD-L1–positive non–small-cell lung cancer. N Engl J Med. 2016;375(19):1823–33. 10.1056/NEJMoa1606774 27718847

[80] Robert C, Ribas A, Schachter J, Arance A, Grob J-J, Mortier L, Daud A, Carlino MS, McNeil CM, Lotem M, Larkin JMG, Lorigan P, Neyns B, Blank CU, Petrella TM, Hamid O, Su S-C, Krepler C, Ibrahim N, Long GV. Pembrolizumab versus ipilimumab in advanced melanoma (KEYNOTE-006): post-hoc 5-year results from an open-label, multicentre, randomised, controlled, phase 3 study. Lancet Oncol. 2019;20(9):1239–51. 10.1016/S1470-2045(19)30388-2 31345627

[81] Robert C, Schachter J, Long GV, Arance A, Grob JJ, Mortier L, Daud A, Carlino MS, McNeil C, Lotem M, Larkin J, Lorigan P, Neyns B, Blank CU, Hamid O, Mateus C, Shapira-Frommer R, Kosh M, Zhou H, Ibrahim N, Ebbinghaus S, Ribas A. Pembrolizumab versus ipilimumab in advanced melanoma. N Engl J Med. 2015;372(26):2521–32. 10.1056/NEJMoa1503093 25891173

[82] Robertson JD, Botwood NA, Rothenberg ML, Schmoll H-J. Phase III trial of FOLFOX plus bevacizumab or cediranib (AZD2171) as first-line treatment of patients with metastatic colorectal cancer: HORIZON III. Clin Colorectal Cancer. 2009;8(1):59–60. 10.3816/CCC.2009.n.010 19203899

[83] Robinson B, Schlumberger M, Wirth LJ, Dutcus CE, Song J, Taylor MH, Kim S-B, Krzyzanowska MK, Capdevila J, Sherman SI, Tahara M. Characterization of tumor size changes over time from the phase 3 study of lenvatinib in thyroid cancer. J Clin Endocrinol Metab. 2016;101(11):4103–9. 10.1210/jc.2015-3989 PMC5095235 27548104

[84] Rosell R, Carcereny E, Gervais R, Vergnenegre A, Massuti B, Felip E, Palmero R, Garcia-Gomez R, Pallares C, Sanchez JM, Porta R, Cobo M, Garrido P, Longo F, Moran T, Insa A, De Marinis F, Corre R, Bover I, Illiano A, Dansin E, de Castro J, Milella M, Reguart N, Altavilla G, Jimenez U, Provencio M, Moreno MA, Terrasa J, Muñoz-Langa J, Valdivia J, Isla D, Domine M, Molinier O, Mazieres J, Baize N, Garcia-Campelo R, Robinet G, Rodriguez-Abreu D, Lopez-Vivanco G, Gebbia V, Ferrera-Delgado L, Bombaron P, Bernabe R, Bearz A, Artal A, Cortesi E, Rolfo C, Sanchez-Ronco M, Drozdowskyj A, Queralt C, de Aguirre I, Ramirez JL, Sanchez JJ, Molina MA, Taron M, Paz-Ares L. Erlotinib versus standard chemotherapy as first-line treatment for European patients with advanced EGFR mutation-positive non-small-cell lung cancer (EURTAC): a multicentre, open-label, randomised phase 3 trial. Lancet Oncol. 2012;13(3):239–46. 10.1016/S1470-2045(11)70393-X 22285168

[85] Rugo HS, Diéras V, Gelmon KA, Finn RS, Slamon DJ, Martin M, Neven P, Shparyk Y, Mori A, Lu DR, Bhattacharyya H, Bartlett CHUANG, Iyer S, Johnston S, Ettl J, Harbeck N. Impact of palbociclib plus letrozole on patient-reported health-related quality of life: results from the PALOMA-2 trial. Ann Oncol. 2018;29(4):888–94. 10.1093/annonc/mdy012 PMC5913649 29360932

[86] Rugo HS, Finn RS, Gelmon K, Joy AA, Harbeck N, Castrellon A, Mukai H, Walshe JM, Mori A, Gauthier E, Lu DR, Bananis E, Martin M, Diéras V. Progression-free survival outcome is independent of objective response in patients with estrogen receptor-positive, human epidermal growth factor receptor 2-negative advanced breast cancer treated with palbociclib plus letrozole compared with letrozole: analysis from PALOMA-2. Clin Breast Cancer. 2020;20(2):e173–80. 10.1016/j.clbc.2019.08.009 31836434

[87] Schachter J, Ribas A, Long GV, Arance A, Grob J-J, Mortier L, Daud A, Carlino MS, McNeil C, Lotem M, Larkin J, Lorigan P, Neyns B, Blank C, Petrella TM, Hamid O, Zhou H, Ebbinghaus S, Ibrahim N, Robert C. Pembrolizumab versus ipilimumab for advanced melanoma: final overall survival results of a multicentre, randomised, open-label phase 3 study (KEYNOTE-006). Lancet. 2017;390(10105):1853–62. 10.1016/S0140-6736(17)31601-X 28822576

[88] Schadendorf D, Larkin J, Wolchok J, Hodi FS, Chiarion-Sileni V, Gonzalez R, Rutkowski P, Grob J-J, Cowey CL, Lao C, Wagstaff J, Callahan MK, Postow MA, Smylie M, Ferrucci PF, Dummer R, Hill A, Taylor F, Sabater J, Walker D, Kotapati S, Abernethy A, Long GV. Health-related quality of life results from the phase III CheckMate 067 study. Eur J Cancer. 2017;82:80–91. 10.1016/j.ejca.2017.05.031 PMC5737813 28651159

[89] Schlumberger M, Elisei R, Müller S, Schöffski P, Brose M, Shah M, Licitra L, Krajewska J, Kreissl MC, Niederle B, Cohen EEW, Wirth L, Ali H, Clary DO, Yaron Y, Mangeshkar M, Ball D, Nelkin B, Sherman S. Overall survival analysis of EXAM, a phase III trial of cabozantinib in patients with radiographically progressive medullary thyroid carcinoma. Ann Oncol. 2017;28(11):2813–9. 10.1093/annonc/mdx479 PMC5834040 29045520

[90] Schlumberger M, Tahara M, Wirth LJ, Robinson B, Brose MS, Elisei R, Habra MA, Newbold K, Shah MH, Hoff AO, Gianoukakis AG, Kiyota N, Taylor MH, Kim S-B, Krzyzanowska MK, Dutcus CE, de las Heras B, Zhu J, Sherman SI. Lenvatinib versus placebo in radioiodine-refractory thyroid cancer. N Engl J Med. 2015;372(7):621–30. 10.1056/NEJMoa1406470 25671254

[91] Schuler M, Yang JC-H, Park K, Kim J-H, Bennouna J, Chen Y-M, Chouaid C, De Marinis F, Feng J-F, Grossi F, Kim D-W, Liu X, Lu S, Strausz J, Vinnyk Y, Wiewrodt R, Zhou C, Wang B, Chand VK, Planchard D, Ou SI, Planchard D, Park K, Schuler M, Yang J, Chand V, Rohr K, Bagnes C, Martin CM, Recondo G, Zarba JJ, Blajman C, Richardet M, McLachlan S-A, Parente P, Underhill C, Crombie C, Mainwaring P, Greil R, Humblet Y, Bustin F, Carestia L, Galdermans D, Lambrechts M, Delval L, Vercauter P, Zhou C, Wang J, Huang C, Lin X, Wu Y, Liu X, Cheng Y, Qin S, Feng J, Huang J, Zhang Y, Lu S, Zereu M, Garicochea B, Zadra CA, Riska H, Alanko T, Cadranel J, Chouaid C, Zalcman G, Sibilot DM, Perol M, Planchard D, Bennouna J, Fournel P, Gervais R, Rotarski M, Coudert B, Schuler M, Thomas M, Wehler T, Faehling M, Keilholz U, Laack E, von Pawel J, Huber R, Dickgreber N, Wiewrodt R, Mark Z, Tehenes S, Strausz J, Sarosi V, Prabhash K, Jain M, Venkatesan S, Sharma L, Dadhich H, Nagarkar RV, Onn A, Gottfried M, Stemmer S, Migliorino MR, Grossi F, Bidoli P, Bearz A, Gridelli C, Milandri C, Platania M, Ceresoli GL, Cruciani G, Delgado FG, Perez JLG, Luna GA, Baca OP, Aerts JGJV, Stigt JA, Dingemans AMC, Herder GJM, Gans SJM, Sánchez JFS, Alvarez Barreda RL, Pantigoso WR, Palomino OLM, Jaskiewicz P, Kazarnowicz A, Serwatowski P, Szczesna A, Jassem J, Lubennikov V, Karaseva N, Orlov S, Ragulin Y, Garrido P, Larriba JLG, Camps C, Campelo RG, Lianes P, Cobo M, Felip E, Kim D-W, Kim S-W, Park K, Kim J-H, Han J-Y, Kim Y-C, Yang C-H, Hsia T-C, Chen Y-M, Tsai Y-H, Chang G-C, Tsao TC-Y, Su W-C, Huang M-S, Ho C-L, Hsieh R-K, Vinnyk Y, Popovych O, Ponomarova O, Bondarenko I, Polishchuk I, Shah R, Mitra S, Popat S, Spicer J, Toy E, Popat S, Talbot T, Brown E, Upadhyay S, Summers Y, Gurtler J, Meza L, Thropay J. Afatinib beyond progression in patients with non-small-cell lung cancer following chemotherapy, erlotinib/gefitinib and afatinib: phase III randomized LUX-Lung 5 trial. Ann Oncol. 2016;27(3):417–23. 10.1093/annonc/mdv597 PMC4769992 26646759

[92] Schuler M, Wu Y-L, Hirsh V, O’Byrne K, Yamamoto N, Mok T, Popat S, Sequist LV, Massey D, Zazulina V, Yang JC-H. First-line afatinib versus chemotherapy in patients with non–small cell lung cancer and common epidermal growth factor receptor gene mutations and brain metastases. J Thor Oncol. 2016;11(3):380–90. 10.1016/j.jtho.2015.11.014 26823294

[93] Shah R, Botteman M, Solem CT, Luo L, Doan J, Cella D, Motzer RJ. A Quality-adjusted time without symptoms or toxicity (Q-TWiST) analysis of nivolumab versus everolimus in advanced renal cell carcinoma (aRCC). Clin Genitourin Cancer. 2019;17(5):356–65.e1. 10.1016/j.clgc.2019.05.010 PMC8262523 31272883

[94] Smith JC, Brooks L, Hoff PM, McWalter G, Dearden S, Morgan SR, Wilson D, Robertson JD, Jürgensmeier JM. KRAS mutations are associated with inferior clinical outcome in patients with metastatic colorectal cancer, but are not predictive for benefit with cediranib. Eur J Cancer. 2013;49(10):2424–32. 10.1016/j.ejca.2013.02.023 23510802

[95] Smith M, De Bono J, Sternberg C, Le Moulec S, Oudard S, De Giorgi U, Krainer M, Bergman A, Hoelzer W, De Wit R, Bögemann M, Saad F, Cruciani G, Thiery-Vuillemin A, Feyerabend S, Miller K, Houédé N, Hussain S, Lam E, Polikoff J, Stenzl A, Mainwaring P, Ramies D, Hessel C, Weitzman A, Fizazi K. Phase III study of cabozantinib in previously treated metastatic castration-resistant prostate cancer: COMET-1. J Clin Oncol. 2016;34(25):3005–13. 10.1200/JCO.2015.65.5597 27400947

[96] Soria J-C, Felip E, Cobo M, Lu S, Syrigos K, Lee KH, Göker E, Georgoulias V, Li W, Isla D, Guclu SZ, Morabito A, Min YJ, Ardizzoni A, Gadgeel SM, Wang B, Chand VK, Goss GD. Afatinib versus erlotinib as second-line treatment of patients with advanced squamous cell carcinoma of the lung (LUX-Lung 8): an open-label randomised controlled phase 3 trial. Lancet Oncol. 2015;16(8):897–907. 10.1016/S1470-2045(15)00006-6 26156651

[97] Tahara M, Brose MS, Wirth LJ, Suzuki T, Miyagishi H, Fujino K, Dutcus CE, Gianoukakis A. Impact of dose interruption on the efficacy of lenvatinib in a phase 3 study in patients with radioiodine-refractory differentiated thyroid cancer. Eur J Cancer. 2019;106:61–8. 10.1016/j.ejca.2018.10.002 30471649

[98] Tahara M, Schlumberger M, Elisei R, Habra MA, Kiyota N, Paschke R, Dutcus CE, Hihara T, McGrath S, Matijevic M, Kadowaki T, Funahashi Y, Sherman SI. Exploratory analysis of biomarkers associated with clinical outcomes from the study of lenvatinib in differentiated cancer of the thyroid. Eur J Cancer. 2017;75:213–21. 10.1016/j.ejca.2017.01.013 28237867

[99] Turner NC, Finn RS, Martin M, Im S-A, DeMichele A, Ettl J, Diéras V, Moulder S, Lipatov O, Colleoni M, Cristofanilli M, Lu DR, Mori A, Giorgetti C, Iyer S, Huang Bartlett C, Gelmon KA. Clinical considerations of the role of palbociclib in the management of advanced breast cancer patients with and without visceral metastases. Ann Oncol. 2018;29(3):669–80. 10.1093/annonc/mdx797 PMC5888946 29342248

[100] Turner NC, Liu Y, Zhu Z, Loi S, Colleoni M, Loibl S, DeMichele A, Harbeck N, André F, Bayar MA, Michiels S, Zhang Z, Giorgetti C, Arnedos M, Bartlett CH, Cristofanilli M. Cyclin E1 Expression and palbociclib efficacy in previously treated hormone receptor–positive metastatic breast cancer. J Clin Oncol. 2019;37(14):1169–78. 10.1200/JCO.18.00925 PMC6506420 30807234

[101] Turner NC, Slamon DJ, Ro J, Bondarenko I, Im S-A, Masuda N, Colleoni M, DeMichele A, Loi S, Verma S, Iwata H, Harbeck N, Loibl S, André F, Theall KP, Huang X, Giorgetti C, Bartlett CH, Cristofanilli M. Overall survival with palbociclib and fulvestrant in advanced breast cancer. N Engl J Med. 2018;379(20):1926–36. 10.1056/NEJMoa1810527 30345905

[102] van Vugt MJH, Stone JA, “Rik” De Greef HJMM, Snyder ES, Lipka L, Turner DC, Chain A, Lala M, Li M, Robey SH, Kondic AG, De Alwis D, Mayawala K, Jain L, Freshwater T. Immunogenicity of pembrolizumab in patients with advanced tumors. J ImmunoTher Cancer. 2019;7(1). 10.1186/s40425-019-0663-4 PMC6686242 31395089

[103] Verma S, Bartlett CH, Schnell P, DeMichele AM, Loi S, Ro J, Colleoni M, Iwata H, Harbeck N, Cristofanilli M, Zhang K, Thiele A, Turner NC, Rugo HS. Palbociclib in combination with fulvestrant in women with hormone receptor‐positive/HER2‐negative advanced metastatic breast cancer: detailed safety analysis from a multicenter, randomized, placebo‐controlled, phase III study (PALOMA‐3). Oncologist. 2016;21(10):1165–75. 10.1634/theoncologist.2016-0097 PMC5061543 27368881

[104] Wolchok JD, Chiarion-Sileni V, Gonzalez R, Rutkowski P, Grob J-J, Cowey CL, Lao CD, Wagstaff J, Schadendorf D, Ferrucci PF, Smylie M, Dummer R, Hill A, Hogg D, Haanen J, Carlino MS, Bechter O, Maio M, Marquez-Rodas I, Guidoboni M, McArthur G, Lebbé C, Ascierto PA, Long GV, Cebon J, Sosman J, Postow MA, Callahan MK, Walker D, Rollin L, Bhore R, Hodi FS, Larkin J. Overall survival with combined nivolumab and ipilimumab in advanced melanoma. N Engl J Med. 2017;377(14):1345–56. 10.1056/NEJMoa1709684 PMC5706778 28889792

[105] Wu Y-L, Sequist LV, Tan E-H, Geater SL, Orlov S, Zhang L, Lee KH, Tsai C-M, Kato T, Barrios CH, Schuler M, Hirsh V, Yamamoto N, O’Byrne K, Boyer M, Mok T, Peil B, Märten A, Yang JC-H, Paz-Ares L, Park K. Afatinib as First-line Treatment of Older Patients With EGFR Mutation-Positive Non-Small-Cell Lung Cancer: Subgroup analyses of the LUX-Lung 3, LUX-Lung 6, and LUX-Lung 7 trials. Clin Lung Cancer. 2018;19(4):e465–79. 10.1016/j.cllc.2018.03.009 29653820

[106] Wu Y-L, Xu C-R, Hu C-P, Feng J, Lu S, Huang Y, Li W, Hou M, Shi JH, Märten A, Fan J, Peil B, Zhou C. 2018;11:8575–87. 10.2147/OTT.S160358 PMC6280988 30584317

[107] Wu Y-L, Zhou C, Hu C-P, Feng J, Lu S, Huang Y, Li W, Hou M, Shi JH, Lee KY, Xu C-R, Massey D, Kim M, Shi Y, Geater SL. Afatinib versus cisplatin plus gemcitabine for first-line treatment of Asian patients with advanced nonsmall-cell lung cancer harbouring EGFR mutations (LUX-Lung 6): an open-label, randomised phase 3 trial. Lancet Oncol. 2014;15(2):213–22. 10.1016/S1470-2045(13)70604-1 24439929

[108] Yamashita T, Kudo M, Ikeda K, Izumi N, Tateishi R, Ikeda M, Aikata H, Kawaguchi Y, Wada Y, Numata K, Inaba Y, Kuromatsu R, Kobayashi M, Okusaka T, Tamai T, Kitamura C, Saito K, Haruna K, Okita K, Kumada H. REFLECT—a phase 3 trial comparing efficacy and safety of lenvatinib to sorafenib for the treatment of unresectable hepatocellular carcinoma: an analysis of Japanese subset. J Gastroenterol. 2020;55(1):113–22. 10.1007/s00535-019-01642-1 PMC6942573 31720835

[109] Yang JC-H, Sequist LV, Zhou C, Schuler M, Geater SL, Mok T, Hu C-P, Yamamoto N, Feng J, O'Byrne K, Lu S, Hirsh V, Huang Y, Sebastian M, Okamoto I, Dickgreber N, Shah R, Märten A, Massey D, Wind S, Wu Y-L. Effect of dose adjustment on the safety and efficacy of afatinib for EGFR mutation-positive lung adenocarcinoma: post hoc analyses of the randomized LUX-Lung 3 and 6 trials. Ann Oncol. 2016;27(11):2103–10. 10.1093/annonc/mdw322 27601237

[110] Yang JC-H, Sequist LV, Geater SL, Tsai C-M, Mok TSK, Schuler M, Yamamoto N, Yu C-J, Ou S-HI, Zhou C, Massey D, Zazulina V, Wu Y-L. Clinical activity of afatinib in patients with advanced non-small-cell lung cancer harbouring uncommon EGFR mutations: a combined post-hoc analysis of LUX-Lung 2, LUX-Lung 3, and LUX-Lung 6. Lancet Oncol. 2015;16(7):830–8. 10.1016/S1470-2045(15)00026-1 26051236

[111] Yang JC-H, Wu Y-L, Schuler M, Sebastian M, Popat S, Yamamoto N, Zhou C, Hu C-P, O'Byrne K, Feng J, Lu S, Huang Y, Geater SL, Lee KY, Tsai C-M, Gorbunova V, Hirsh V, Bennouna J, Orlov S, Mok T, Boyer M, Su W-C, Lee KH, Kato T, Massey D, Shahidi M, Zazulina V, Sequist LV. Afatinib versus cisplatin-based chemotherapy for EGFR mutation-positive lung adenocarcinoma (LUX-Lung 3 and LUX-Lung 6): analysis of overall survival data from two randomised, phase 3 trials. Lancet Oncol. 2015;16(2):141–51. 10.1016/S1470-2045(14)71173-8 25589191

[112] Yao JC, Fazio N, Singh S, Buzzoni R, Carnaghi C, Wolin E, Tomasek J, Raderer M, Lahner H, Voi M, Pacaud LB, Rouyrre N, Sachs C, Valle JW, Fave GD, Van Cutsem E, Tesselaar M, Shimada Y, Oh D-Y, Strosberg J, Kulke MH, Pavel ME. Everolimus for the treatment of advanced, non-functional neuroendocrine tumours of the lung or gastrointestinal tract (RADIANT-4): a randomised, placebo-controlled, phase 3 study. Lancet. 2016;387(10022):968–77. 10.1016/S0140-6736(15)00817-X PMC6063317 26703889

[113] Golder S, Loke YK, Wright K, Norman G. Reporting of adverse events in published and unpublished studies of health care interventions: a systematic review. Plos Med. 2016;13. 10.1371/journal.pmed.1002127. 10.1371/journal.pmed.1002127 PMC5029817 27649528

[114] Schulz KF, Altman DG, Moher D. CONSORT 2010 Statement: updated guidelines for reporting parallel group randomised trials. BMJ. 2010;340. 10.1136/bmj.c332. 10.1136/bmj.c332 PMC2844940 20332509

[115] Ioannidis JPA, Evans SJW, Gøtzsche PC, O’Neill RT, Altman DG, Schulz K (2004). Better reporting of harms in randomized trials: an extension of the CONSORT statement. Ann Intern Med.

[116] Phillips R, Hazell L, Sauzet O, Cornelius V (2019). Analysis and reporting of adverse events in randomised controlled trials: a review. BMJ Open.

[117] Transparency International, TranspariMED (2017). CLINICAL TRIAL TRANSPARENCY - A guide for policy makers.

[118] Maruani A, Boutron I, Baron G, Ravaud P (2014). Impact of sending email reminders of the legal requirement for posting results on ClinicalTrials.gov: cohort embedded pragmatic randomized controlled trial. BMJ.

[119] European Medicines Agency (2020). Response to the Institute for Quality and Efficiency in Health Care (IQWiG) and the Cochrane Collaboration on transparency of COVID-19-related activities.

