# Supplementary material for: Reporting of harms in oncological clinical study reports submitted to the European Medicines Agency compared to trial registries and publications—a methodological review
Source: BMC Med. 2021 Apr 8;19:88. doi: 10.1186/s12916-021-01955-0 (PMC8028762; doi:10.1186/s12916-021-01955-0)
Supplement: Supplementary file 1 — Additional file 1. Characteristics of included trials. [file 12916_2021_1955_MOESM1_ESM.docx]

**Information about included trials**

| **Trial name** | **Registration number** | **Drug name** | **Phase** | **Pharmaceutical company** | **Type of cancer** |
| --- | --- | --- | --- | --- | --- |
| LUX-Head & Neck 1 | NCT01345682 | Afatinib | 3 | Boehringer Ingelheim | Head and Neck Squamous Cell carcinoma |
| LUX-Lung 5 | NCT01085136 | Afatinib | 3 | Boehringer Ingelheim | Non-small cell lung cancer |
| LUX-Lung 6 | NCT01121393 | Afatinib | 3 | Boehringer Ingelheim | Non-small cell lung cancer (non-squamous with an EGFR-activating mutation) |
| LUX-Lung 8 | NCT01523587 | Afatinib | 3 | Boehringer Ingelheim | Non-small cell lung cancer (squamous cell carcinoma) |
| LUX-LUNG 1 | NCT00656136 | Afatinib | 2/3 | Boehringer Ingelheim | Non-small cell lung cancer |
|  | NCT00514943 | Afatinib | 2 | Boehringer Ingelheim | Head and Neck Squamous Cell carcinoma |
| ATLAS | NCT00257608 | Bevacizumab | 3 | Roche | Non-small cell lung cancer |
| EURTAC | NCT00446225 | Bevacizumab | 3 | Roche | Non-small cell lung cancer with an EGFR-activating mutation |
|  | JapicCTI-111390 | Bevacizumab | 2 | Roche | Non-small cell lung cancer |
|  | NCT00531960 | Bevacizumab | 2 | Roche | Non-small cell lung cancer |
|  | NCT00095225 | Bevacizumab | 2 | Roche | Non-small cell lung cancer |
| BeTa | NCT00130728 | Bevacizumab | 3 | Roche | Non-small cell lung cancer |
| EXAM | NCT00704730 | Cabozantinib | 3 | Exelixis | Medullary Thyroid Cancer |
| METEOR | NCT01865747 | Cabozantinib | 3 | Exelixis | Renal Cell Carcinoma |
| COMET-2 | NCT01522443 | Cabozantinib | 3 | Exelixis | Prostate Cancer |
| COMET-1 | NCT01605227 | Cabozantinib | 3 | Exelixis | Prostate Cancer |
| ICON6 | NCT00532194 | Cediranib | 3 | AstraZeneca | Ovarian Cancer |
| HORIZON III | NCT00384176 | Cediranib | 2/3 | AstraZeneca | Colorectal Cancer |
|  | NCT00423332 | Cediranib | 2 | AstraZeneca | Renal Cell Carcinoma |
| HORIZON II | NCT00399035 | Cediranib | 3 | AstraZeneca | Colorectal Cancer |
|  | NCT00795340 | Cediranib | 3 | NCIC Clinical Trials Group | Non-small cell lung cancer |
|  | NCT00245154 | Cediranib | 2/3 | NCIC Clinical Trials Group | Non-small cell lung cancer |
| REGAL | NCT00777153 | Cediranib | 3 | AstraZeneca | Glioblastoma |
|  | NCT00556712 | Erlotinib | 3 | Roche | Non-small cell lung cancer |
| RADIANT-4 | NCT01524783 | Everolimus | 3 | Novartis | Neuro-Endocrin Tumor (gastro-intestinal or lung origin) |
|  | NCT01529112 | Lenvatinib | 2 | Eisai | Non-small cell lung cancer (non-squamous) |
|  | NCT01137604 | Lenvatinib | 2 | Eisai | Glioma |
| SELECT | NCT01321554 | Lenvatinib | 3 | Eisai | Differentiated Thyroid Cancer |
|  | NCT01761266 | Lenvatinib | 3 | Eisai | Hepatocellular Carcinoma |
| CheckMate 025 | NCT01668784 | Nivolumab | 3 | Bristol-Myers Squibb | Renal Cell Carcinoma (Clear-cell) |
| CheckMate 057 | NCT01673867 | Nivolumab | 3 | Bristol-Myers Squibb | Non-small cell lung cancer (non-squamous) |
| CheckMate 067 | NCT01844505 | Nivolumab | 3 | Bristol-Myers Squibb | Melanoma |
| Checkmate 069 | NCT01927419 | Nivolumab | 2 | Bristol-Myers Squibb | Melanoma |
|  | NCT00913835 | Olaratumab | 2 | ImClone Systems | Ovarian Cancer |
|  | NCT00918203 | Olaratumab | 2 | ImClone Systems | Non-small cell lung cancer |
|  | NCT01204710 | Olaratumab | 2 | ImClone Systems | Prostate Cancer |
| PALOMA-2 | NCT01740427 | Palbociclib | 3 | Pfizer | Breast Cancer |
| PALOMA-3 | NCT01942135 | Palbociclib | 3 | Pfizer | Breast Cancer |
| PALOMA-4 | NCT02297438 | Palbociclib | 3 | Pfizer | Breast Cancer |
| Keynote-006 | NCT01866319 | Pembrolizumab | 3 | Merck | Melanoma |
| Keynote-010 | NCT01905657 | Pembrolizumab | 2/3 | Merck | Non-small cell lung cancer |
| Keynote-024 | NCT02142738 | Pembrolizumab | 3 | Merck | Non-small cell lung cancer |

**List of publications used for extraction of data on harms**

**1200.28**

No publications identified

**LUX-Head&Neck 1**

1. Machiels J-PH, Haddad RI, Fayette J, et al. Afatinib versus methotrexate as second-line treatment in patients with recurrent or metastatic squamous-cell carcinoma of the head and neck progressing on or after platinum-based therapy (LUX-Head & Neck 1): an open-label, randomised phase 3 trial. Lancet Oncol 2015;16:583–94. doi:10.1016/S1470-2045(15)70124-5
2. Clement PM, Gauler T, Machiels JP, et al. Afatinib versus methotrexate in older patients with second-line recurrent and/or metastatic head and neck squamous cell carcinoma: subgroup analysis of the LUX-Head & Neck 1 trial. Ann Oncol 2016;27:1585–93. doi:10.1093/annonc/mdw151
3. Cohen EEW, Licitra LF, Burtness B, et al. Biomarkers predict enhanced clinical outcomes with afatinib versus methotrexate in patients with second-line recurrent and/or metastatic head and neck cancer. Ann Oncol 2017;28:2526–32. doi:10.1093/annonc/mdx344

**LUX-LUNG 1**

1. Miller VA, Hirsh V, Cadranel J, et al. Afatinib versus placebo for patients with advanced, metastatic non-small-cell lung cancer after failure of erlotinib, gefitinib, or both, and one or two lines of chemotherapy (LUX-Lung 1): a phase 2b/3 randomised trial. Lancet Oncol 2012;13:528–38. doi:10.1016/S1470-2045(12)70087-6

**LUX-LUNG 5**

1. Schuler M, Yang JC-H, Park K, et al. Afatinib beyond progression in patients with non-small-cell lung cancer following chemotherapy, erlotinib/gefitinib and afatinib: phase III randomized LUX-Lung 5 trial. Ann Oncol 2016;27:417–23. doi:10.1093/annonc/mdv597

**LUX-LUNG 6**

1. Wu Y-L, Sequist LV, Tan E-H, et al. Afatinib as First-line Treatment of Older Patients With EGFR Mutation-Positive Non-Small-Cell Lung Cancer: Subgroup Analyses of the LUX-Lung 3, LUX-Lung 6, and LUX-Lung 7 Trials. Clin Lung Cancer 2018;19:e465–79. doi:10.1016/j.cllc.2018.03.009
2. Wu Y-L, Zhou C, Hu C-P, et al. Afatinib versus cisplatin plus gemcitabine for first-line treatment of Asian patients with advanced non-small-cell lung cancer harbouring EGFR mutations (LUX-Lung 6): an open-label, randomised phase 3 trial. Lancet Oncol 2014;15:213–22. doi:10.1016/S1470-2045(13)70604-1
3. Yang JC-H, Wu Y-L, Schuler M, et al. Afatinib versus cisplatin-based chemotherapy for EGFR mutation-positive lung adenocarcinoma (LUX-Lung 3 and LUX-Lung 6): analysis of overall survival data from two randomised, phase 3 trials. Lancet Oncol 2015;16:141–51. doi:10.1016/S1470-2045(14)71173-8
4. Wu Y-L, Xu C-R, Hu C-P, et al. Afatinib versus gemcitabine/cisplatin for first-line treatment of Chinese patients with advanced non-small-cell lung cancer harboring EGFR mutations: subgroup analysis of the LUX-Lung 6 trial. Onco Targets Ther 2018;11:8575–87. doi:10.2147/OTT.S160358
5. Yang JC-H, Sequist LV, Geater SL, et al. Clinical activity of afatinib in patients with advanced non-small-cell lung cancer harbouring uncommon EGFR mutations: a combined post-hoc analysis of LUX-Lung 2, LUX-Lung 3, and LUX-Lung 6. Lancet Oncol 2015;16:830–8. doi:10.1016/S1470-2045(15)00026-1
6. Yang JC-H, Sequist LV, Zhou C, et al. Effect of dose adjustment on the safety and efficacy of afatinib for EGFR mutation-positive lung adenocarcinoma: post hoc analyses of the randomized LUX-Lung 3 and 6 trials. Ann Oncol 2016;27:2103–10. doi:10.1093/annonc/mdw322
7. Schuler M, Wu Y-L, Hirsh V, et al. First-Line Afatinib versus Chemotherapy in Patients with Non-Small Cell Lung Cancer and Common Epidermal Growth Factor Receptor Gene Mutations and Brain Metastases. J Thorac Oncol 2016;11:380–90. doi:10.1016/j.jtho.2015.11.014

**LUX-LUNG 8**

1. Soria J-C, Felip E, Cobo M, et al. Afatinib versus erlotinib as second-line treatment of patients with advanced squamous cell carcinoma of the lung (LUX-Lung 8): an open-label randomised controlled phase 3 trial. Lancet Oncol 2015;16:897–907. doi:10.1016/S1470-2045(15)00006-6
2. Lu S, Li W, Zhou C, et al. Afatinib vs erlotinib for second-line treatment of Chinese patients with advanced squamous cell carcinoma of the lung. Onco Targets Ther 2018;11:8565–73. doi:10.2147/OTT.S161506
3. Goss GD, Felip E, Cobo M, et al. Association of ERBB Mutations With Clinical Outcomes of Afatinib- or Erlotinib-Treated Patients With Lung Squamous Cell Carcinoma: Secondary Analysis of the LUX-Lung 8 Randomized Clinical Trial. JAMA Oncol 2018;4:1189–97. doi:10.1001/jamaoncol.2018.0775
4. Gadgeel S, Goss G, Soria J-C, et al. Evaluation of the VeriStrat® serum protein test in patients with advanced squamous cell carcinoma of the lung treated with second-line afatinib or erlotinib in the phase III LUX-Lung 8 study. Lung Cancer 2017;109:101–8. doi:10.1016/j.lungcan.2017.05.010

**ATLAS**

1. Johnson BE, Kabbinavar F, Fehrenbacher L, et al. ATLAS: randomized, double-blind, placebo-controlled, phase IIIB trial comparing bevacizumab therapy with or without erlotinib, after completion of chemotherapy, with bevacizumab for first-line treatment of advanced non-small-cell lung cancer. J Clin Oncol 2013;31:3926–34. doi:10.1200/JCO.2012.47.3983

**BeTa**

1. Herbst RS, Ansari R, Bustin F, et al. Efficacy of bevacizumab plus erlotinib versus erlotinib alone in advanced non-small-cell lung cancer after failure of standard first-line chemotherapy (BeTa): a double-blind, placebo-controlled, phase 3 trial. Lancet 2011;377:1846–54. doi:10.1016/S0140-6736(11)60545-X

**BO20571**

1. Ciuleanu T, Tsai C-M, Tsao C-J, et al. A phase II study of erlotinib in combination with bevacizumab versus chemotherapy plus bevacizumab in the first-line treatment of advanced non-squamous non-small cell lung cancer. Lung Cancer 2013;82:276–81. doi:10.1016/j.lungcan.2013.08.002

**EURTAC**

1. Karachaliou N, Mayo-de las Casas C, Queralt C, et al. Association of EGFR L858R Mutation in Circulating Free DNA With Survival in the EURTAC Trial. JAMA Oncol 2015;1:149–57. doi:10.1001/jamaoncol.2014.257
2. Rosell R, Carcereny E, Gervais R, et al. Erlotinib versus standard chemotherapy as first-line treatment for European patients with advanced EGFR mutation-positive non-small-cell lung cancer (EURTAC): a multicentre, open-label, randomised phase 3 trial. Lancet Oncol 2012;13:239–46. doi:10.1016/S1470-2045(11)70393-X
3. Karachaliou N, Gimenez-Capitan A, Drozdowskyj A, et al. ROR1 as a novel therapeutic target for EGFR-mutant non-small-cell lung cancer patients with the EGFR T790M mutation. Transl Lung Cancer Res 2014;3:122–30. doi:10.3978/j.issn.2218-6751.2014.03.02
4. Costa C, Molina MA, Drozdowskyj A, et al. The impact of EGFR T790M mutations and BIM mRNA expression on outcome in patients with EGFR-mutant NSCLC treated with erlotinib or chemotherapy in the randomized phase III EURTAC trial. Clin Cancer Res 2014;20:2001–10. doi:10.1158/1078-0432.CCR-13-2233

**JO25567**

No publications identified

**OSI2950g**

No publications identified

**COMET-1**

1. Smith M, De Bono J, Sternberg C, et al. Phase III Study of Cabozantinib in Previously Treated Metastatic Castration-Resistant Prostate Cancer: COMET-1. J Clin Oncol 2016;34:3005–13. doi:10.1200/JCO.2015.65.5597

**COMET-2**

1. Dueck AC, Scher HI, Bennett AV, et al. Assessment of Adverse Events From the Patient Perspective in a Phase 3 Metastatic Castration-Resistant Prostate Cancer Clinical Trial. JAMA Oncol Published Online First: 26 September 2019. doi:10.1001/jamaoncol.2019.3332
2. Basch EM, Scholz M, de Bono JS, et al. Cabozantinib Versus Mitoxantrone-prednisone in Symptomatic Metastatic Castration-resistant Prostate Cancer: A Randomized Phase 3 Trial with a Primary Pain Endpoint. Eur Urol 2019;75:929–37. doi:10.1016/j.eururo.2018.11.033

**EXAM**

1. Elisei R, Schlumberger MJ, Müller SP, et al. Cabozantinib in progressive medullary thyroid cancer. J Clin Oncol 2013;31:3639–46. doi:10.1200/JCO.2012.48.4659
2. Schlumberger M, Elisei R, Müller S, et al. Overall survival analysis of EXAM, a phase III trial of cabozantinib in patients with radiographically progressive medullary thyroid carcinoma. Ann Oncol 2017;28:2813–9. doi:10.1093/annonc/mdx479

**METEOR**

1. Choueiri TK, Escudier B, Powles T, et al. Cabozantinib versus everolimus in advanced renal cell carcinoma (METEOR): final results from a randomised, open-label, phase 3 trial. Lancet Oncol 2016;17:917–27. doi:10.1016/S1470-2045(16)30107-3
2. Choueiri TK, Escudier B, Powles T, et al. Cabozantinib versus Everolimus in Advanced Renal-Cell Carcinoma. N Engl J Med 2015;373:1814–23. doi:10.1056/NEJMoa1510016
3. Escudier B, Powles T, Motzer RJ, et al. Cabozantinib, a New Standard of Care for Patients With Advanced Renal Cell Carcinoma and Bone Metastases? Subgroup Analysis of the METEOR Trial. J Clin Oncol 2018;36:765–72. doi:10.1200/JCO.2017.74.7352
4. Cella D, Escudier B, Tannir NM, et al. Quality of Life Outcomes for Cabozantinib Versus Everolimus in Patients With Metastatic Renal Cell Carcinoma: METEOR Phase III Randomized Trial. J Clin Oncol 2018;36:757–64. doi:10.1200/JCO.2017.75.2170

**D8480C00030**

1. Mulders P, Hawkins R, Nathan P, et al. Cediranib monotherapy in patients with advanced renal cell carcinoma: results of a randomised phase II study. Eur J Cancer 2012;48:527–37. doi:10.1016/j.ejca.2011.12.022

**HORIZON II**

1. Smith JC, Brooks L, Hoff PM, et al. KRAS mutations are associated with inferior clinical outcome in patients with metastatic colorectal cancer, but are not predictive for benefit with cediranib. Eur J Cancer 2013;49:2424–32. doi:10.1016/j.ejca.2013.02.023

**HORIZON III**

1. Robertson JD, Botwood NA, Rothenberg ML, et al. Phase III trial of FOLFOX plus bevacizumab or cediranib (AZD2171) as first-line treatment of patients with metastatic colorectal cancer: HORIZON III. Clin Colorectal Cancer 2009;8:59–60. doi:10.3816/CCC.2009.n.010

**ICON 6**

1. Ledermann JA, Embleton AC, Raja F, *et al.* Cediranib in patients with relapsed platinum-sensitive ovarian cancer (ICON6): a randomised, double-blind, placebo-controlled phase 3 trial. *Lancet* 2016;387:1066–74. doi:[10.1016/S0140-6736(15)01167-8](https://doi.org/10.1016/S0140-6736(15)01167-8)

**NCIC CTG BR.24**

1. Goss GD, Arnold A, Shepherd FA, *et al.* Randomized, double-blind trial of carboplatin and paclitaxel with either daily oral cediranib or placebo in advanced non-small-cell lung cancer: NCIC clinical trials group BR24 study. *J Clin Oncol* 2010;**28**:49–55. doi:[10.1200/JCO.2009.22.9427](https://doi.org/10.1200/JCO.2009.22.9427)

**NCIC CTG BR.29**

1. Laurie SA, Solomon BJ, Seymour L, *et al.* Randomised, double-blind trial of carboplatin and paclitaxel with daily oral cediranib or placebo in patients with advanced non-small cell lung cancer: NCIC Clinical Trials Group study BR29. *Eur J Cancer* 2014;**50**:706–12. doi:[10.1016/j.ejca.2013.11.032](https://doi.org/10.1016/j.ejca.2013.11.032)

**REGAL**

1. Batchelor TT, Mulholland P, Neyns B, *et al.* Phase III randomized trial comparing the efficacy of cediranib as monotherapy, and in combination with lomustine, versus lomustine alone in patients with recurrent glioblastoma. *J Clin Oncol* 2013;**31**:3212–8. doi:[10.1200/JCO.2012.47.2464](https://doi.org/10.1200/JCO.2012.47.2464)

**BO18192**

1. Cappuzzo F, Ciuleanu T, Stelmakh L, et al. Erlotinib as maintenance treatment in advanced non-small-cell lung cancer: a multicentre, randomised, placebo-controlled phase 3 study. Lancet Oncol 2010;11:521–9. doi:10.1016/S1470-2045(10)70112-1

**RADIANT-4**

1. Yao JC, Fazio N, Singh S, et al. Everolimus for the treatment of advanced, non-functional neuroendocrine tumours of the lung or gastrointestinal tract (RADIANT-4): a randomised, placebo-controlled, phase 3 study. Lancet 2016;387:968–77. doi:10.1016/S0140-6736(15)00817-X
2. Fazio N, Buzzoni R, Delle Fave G, et al. Everolimus in advanced, progressive, well-differentiated, non-functional neuroendocrine tumors: RADIANT-4 lung subgroup analysis. Cancer Sci 2018;109:174–81. doi:10.1111/cas.13427
3. Pavel ME, Singh S, Strosberg JR, et al. Health-related quality of life for everolimus versus placebo in patients with advanced, non-functional, well-differentiated gastrointestinal or lung neuroendocrine tumours (RADIANT-4): a multicentre, randomised, double-blind, placebo-controlled, phase 3 trial. Lancet Oncol 2017;18:1411–22. doi:10.1016/S1470-2045(17)30471-0
4. Buzzoni R, Carnaghi C, Strosberg J, et al. Impact of prior therapies on everolimus activity: an exploratory analysis of RADIANT-4. Onco Targets Ther 2017;10:5013–30. doi:10.2147/OTT.S142087

**E7080-703**

No publication identified

**E7080-G000-203**

No publication identified

**E7080-G000-304**

1. Kudo M, Finn RS, Qin S, et al. Lenvatinib versus sorafenib in first-line treatment of patients with unresectable hepatocellular carcinoma: a randomised phase 3 non-inferiority trial. Lancet 2018;391:1163–73. doi:10.1016/S0140-6736(18)30207-1
2. Yamashita T, Kudo M, Ikeda K, et al. REFLECT-a phase 3 trial comparing efficacy and safety of lenvatinib to sorafenib for the treatment of unresectable hepatocellular carcinoma: an analysis of Japanese subset. J Gastroenterol 2020;55:113–22. doi:10.1007/s00535-019-01642-1
3. Evans TRJ, Kudo M, Finn RS, et al. Urine protein:creatinine ratio vs 24-hour urine protein for proteinuria management: analysis from the phase 3 REFLECT study of lenvatinib vs sorafenib in hepatocellular carcinoma. Br J Cancer 2019;121:218–21. doi:10.1038/s41416-019-0506-6

**SELECT**

1. Robinson B, Schlumberger M, Wirth LJ, et al. Characterization of Tumor Size Changes Over Time From the Phase 3 Study of Lenvatinib in Thyroid Cancer. J Clin Endocrinol Metab 2016;101:4103–9. doi:10.1210/jc.2015-3989
2. Tahara M, Schlumberger M, Elisei R, et al. Exploratory analysis of biomarkers associated with clinical outcomes from the study of lenvatinib in differentiated cancer of the thyroid. Eur J Cancer 2017;75:213–21. doi:10.1016/j.ejca.2017.01.013
3. Tahara M, Brose MS, Wirth LJ, et al. Impact of dose interruption on the efficacy of lenvatinib in a phase 3 study in patients with radioiodine-refractory differentiated thyroid cancer. Eur J Cancer 2019;106:61–8. doi:10.1016/j.ejca.2018.10.002
4. Schlumberger M, Tahara M, Wirth LJ, et al. Lenvatinib versus placebo in radioiodine-refractory thyroid cancer. N Engl J Med 2015;372:621–30. doi:10.1056/NEJMoa1406470
5. Kiyota N, Schlumberger M, Muro K, et al. Subgroup analysis of Japanese patients in a phase 3 study of lenvatinib in radioiodine-refractory differentiated thyroid cancer. Cancer Sci 2015;106:1714–21. doi:10.1111/cas.12826

**Checkmate-025**

1. Shah R, Botteman M, Solem CT, et al. A Quality-adjusted Time Without Symptoms or Toxicity (Q-TWiST) Analysis of Nivolumab Versus Everolimus in Advanced Renal Cell Carcinoma (aRCC). Clin Genitourin Cancer 2019;17:356-365.e1. doi:10.1016/j.clgc.2019.05.010
2. Escudier B, Sharma P, McDermott DF, et al. CheckMate 025 Randomized Phase 3 Study: Outcomes by Key Baseline Factors and Prior Therapy for Nivolumab Versus Everolimus in Advanced Renal Cell Carcinoma. Eur Urol 2017;72:962–71. doi:10.1016/j.eururo.2017.02.010
3. Motzer RJ, Escudier B, McDermott DF, et al. Nivolumab versus Everolimus in Advanced Renal-Cell Carcinoma. N Engl J Med 2015;373:1803–13. doi:10.1056/NEJMoa1510665
4. Cella D, Grünwald V, Nathan P, et al. Quality of life in patients with advanced renal cell carcinoma given nivolumab versus everolimus in CheckMate 025: a randomised, open-label, phase 3 trial. Lancet Oncol 2016;17:994–1003. doi:10.1016/S1470-2045(16)30125-5
5. Escudier B, Motzer RJ, Sharma P, et al. Treatment Beyond Progression in Patients with Advanced Renal Cell Carcinoma Treated with Nivolumab in CheckMate 025. Eur Urol 2017;72:368–76. doi:10.1016/j.eururo.2017.03.037

**Checkmate-057**

1. Reck M, Brahmer J, Bennett B, et al. Evaluation of health-related quality of life and symptoms in patients with advanced non-squamous non-small cell lung cancer treated with nivolumab or docetaxel in CheckMate 057. Eur J Cancer 2018;102:23–30. doi:10.1016/j.ejca.2018.05.005
2. Borghaei H, Paz-Ares L, Horn L, et al. Nivolumab versus Docetaxel in Advanced Nonsquamous Non-Small-Cell Lung Cancer. N Engl J Med 2015;373:1627–39. doi:10.1056/NEJMoa1507643

**Checkmate-067**

1. Larkin J, Chiarion-Sileni V, Gonzalez R, et al. Combined Nivolumab and Ipilimumab or Monotherapy in Untreated Melanoma. N Engl J Med 2015;373:23–34. doi:10.1056/NEJMoa1504030
2. Larkin J, Chiarion-Sileni V, Gonzalez R, et al. Five-Year Survival with Combined Nivolumab and Ipilimumab in Advanced Melanoma. N Engl J Med 2019;381:1535–46. doi:10.1056/NEJMoa1910836
3. Schadendorf D, Larkin J, Wolchok J, et al. Health-related quality of life results from the phase III CheckMate 067 study. Eur J Cancer 2017;82:80–91. doi:10.1016/j.ejca.2017.05.031
4. Hodi FS, Chiarion-Sileni V, Gonzalez R, et al. Nivolumab plus ipilimumab or nivolumab alone versus ipilimumab alone in advanced melanoma (CheckMate 067): 4-year outcomes of a multicentre, randomised, phase 3 trial. Lancet Oncol 2018;19:1480–92. doi:10.1016/S1470-2045(18)30700-9
5. Wolchok JD, Chiarion-Sileni V, Gonzalez R, et al. Overall Survival with Combined Nivolumab and Ipilimumab in Advanced Melanoma. N Engl J Med 2017;377:1345–56. doi:10.1056/NEJMoa1709684

**Checkmate-069**

1. Hodi FS, Chesney J, Pavlick AC, et al. Combined nivolumab and ipilimumab versus ipilimumab alone in patients with advanced melanoma: 2-year overall survival outcomes in a multicentre, randomised, controlled, phase 2 trial. Lancet Oncol 2016;17:1558–68. doi:10.1016/S1470-2045(16)30366-7
2. Postow MA, Chesney J, Pavlick AC, et al. Nivolumab and ipilimumab versus ipilimumab in untreated melanoma. N Engl J Med 2015;372:2006–17. doi:10.1056/NEJMoa1414428

**I5B-IE-JGDA**

1. McGuire WP, Penson RT, Gore M, et al. Randomized phase II study of the PDGFRα antibody olaratumab plus liposomal doxorubicin versus liposomal doxorubicin alone in patients with platinum-refractory or platinum-resistant advanced ovarian cancer. BMC Cancer 2018;18:1292. doi:10.1186/s12885-018-5198-4

**I5B-IE-JGDB**

No publication identified

**I5B-IE-JGDD**

No publication identified

**PALOMA-2**

1. Rugo HS, Diéras V, Gelmon KA, et al. Impact of palbociclib plus letrozole on patient-reported health-related quality of life: results from the PALOMA-2 trial. Ann Oncol 2018;29:888–94. doi:10.1093/annonc/mdy012
2. Finn RS, Martin M, Rugo HS, et al. Palbociclib and Letrozole in Advanced Breast Cancer. N Engl J Med 2016;375:1925–36. doi:10.1056/NEJMoa1607303
3. Mukai H, Shimizu C, Masuda N, et al. Palbociclib in combination with letrozole in patients with estrogen receptor-positive, human epidermal growth factor receptor 2-negative advanced breast cancer: PALOMA-2 subgroup analysis of Japanese patients. Int J Clin Oncol 2019;24:274–87. doi:10.1007/s10147-018-1353-9
4. Rugo HS, Finn RS, Gelmon K, et al. Progression-free Survival Outcome Is Independent of Objective Response in Patients With Estrogen Receptor-positive, Human Epidermal Growth Factor Receptor 2-negative Advanced Breast Cancer Treated With Palbociclib Plus Letrozole Compared With Letrozole: Analysis From PALOMA-2. Clin Breast Cancer Published Online First: 5 September 2019. doi:10.1016/j.clbc.2019.08.009

**PALOMA-3**

1. Turner NC, Finn RS, Martin M, et al. Clinical considerations of the role of palbociclib in the management of advanced breast cancer patients with and without visceral metastases. Ann Oncol 2018;29:669–80. doi:10.1093/annonc/mdx797
2. Turner NC, Liu Y, Zhu Z, et al. Cyclin E1 Expression and Palbociclib Efficacy in Previously Treated Hormone Receptor-Positive Metastatic Breast Cancer. J Clin Oncol 2019;37:1169–78. doi:10.1200/JCO.18.00925
3. Turner NC, Slamon DJ, Ro J, et al. Overall Survival with Palbociclib and Fulvestrant in Advanced Breast Cancer. N Engl J Med 2018;379:1926–36. doi:10.1056/NEJMoa1810527
4. Loibl S, Turner NC, Ro J, et al. Palbociclib Combined with Fulvestrant in Premenopausal Women with Advanced Breast Cancer and Prior Progression on Endocrine Therapy: PALOMA-3 Results. Oncologist 2017;22:1028–38. doi:10.1634/theoncologist.2017-0072
5. Masuda N, Inoue K, Nakamura R, et al. Palbociclib in combination with fulvestrant in patients with hormone receptor-positive, human epidermal growth factor receptor 2-negative advanced breast cancer: PALOMA-3 subgroup analysis of Japanese patients. Int J Clin Oncol 2019;24:262–73. doi:10.1007/s10147-018-1359-3
6. Verma S, Bartlett CH, Schnell P, et al. Palbociclib in Combination With Fulvestrant in Women With Hormone Receptor-Positive/HER2-Negative Advanced Metastatic Breast Cancer: Detailed Safety Analysis From a Multicenter, Randomized, Placebo-Controlled, Phase III Study (PALOMA-3). Oncologist 2016;21:1165–75. doi:10.1634/theoncologist.2016-0097
7. Turner NC, Ro J, André F, et al. Palbociclib in Hormone-Receptor-Positive Advanced Breast Cancer. N Engl J Med 2015;373:209–19. doi:10.1056/NEJMoa1505270
8. Cristofanilli M, DeMichele A, Giorgetti C, et al. Predictors of prolonged benefit from palbociclib plus fulvestrant in women with endocrine-resistant hormone receptor-positive/human epidermal growth factor receptor 2-negative metastatic breast cancer in PALOMA-3. Eur J Cancer 2018;104:21–31. doi:10.1016/j.ejca.2018.08.011
9. Rugo HS, Finn RS, Gelmon K, et al. Progression-free Survival Outcome Is Independent of Objective Response in Patients With Estrogen Receptor-positive, Human Epidermal Growth Factor Receptor 2-negative Advanced Breast Cancer Treated With Palbociclib Plus Letrozole Compared With Letrozole: Analysis From PALOMA-2. Clin Breast Cancer Published Online First: 5 September 2019. doi:10.1016/j.clbc.2019.08.009
10. Harbeck N, Iyer S, Turner N, et al. Quality of life with palbociclib plus fulvestrant in previously treated hormone receptor-positive, HER2-negative metastatic breast cancer: patient-reported outcomes from the PALOMA-3 trial. Ann Oncol 2016;27:1047–54. doi:10.1093/annonc/mdw139

**PALOMA-4**

No publications identified

**Keynote-006**

1. Carlino MS, Long GV, Schadendorf D, et al. Outcomes by line of therapy and programmed death ligand 1 expression in patients with advanced melanoma treated with pembrolizumab or ipilimumab in KEYNOTE-006: A randomised clinical trial. Eur J Cancer 2018;101:236–43. doi:10.1016/j.ejca.2018.06.034
2. Petrella TM, Robert C, Richtig E, et al. Patient-reported outcomes in KEYNOTE-006, a randomised study of pembrolizumab versus ipilimumab in patients with advanced melanoma. Eur J Cancer 2017;86:115–24. doi:10.1016/j.ejca.2017.08.032
3. Schachter J, Ribas A, Long GV, et al. Pembrolizumab versus ipilimumab for advanced melanoma: final overall survival results of a multicentre, randomised, open-label phase 3 study (KEYNOTE-006). Lancet 2017;390:1853–62. doi:10.1016/S0140-6736(17)31601-X
4. Robert C, Schachter J, Long GV, et al. Pembrolizumab versus Ipilimumab in Advanced Melanoma. N Engl J Med 2015;372:2521–32. doi:10.1056/NEJMoa1503093
5. Robert C, Ribas A, Schachter J, et al. Pembrolizumab versus ipilimumab in advanced melanoma (KEYNOTE-006): post-hoc 5-year results from an open-label, multicentre, randomised, controlled, phase 3 study. Lancet Oncol 2019;20:1239–51. doi:10.1016/S1470-2045(19)30388-2

**Keynote-010**

1. Barlesi F, Garon EB, Kim D-W, et al. Health-Related Quality of Life in KEYNOTE-010: a Phase II/III Study of Pembrolizumab Versus Docetaxel in Patients With Previously Treated Advanced, Programmed Death Ligand 1-Expressing NSCLC. J Thorac Oncol 2019;14:793–801. doi:10.1016/j.jtho.2019.01.016
2. Herbst RS, Baas P, Kim D-W, et al. Pembrolizumab versus docetaxel for previously treated, PD-L1-positive, advanced non-small-cell lung cancer (KEYNOTE-010): a randomised controlled trial. Lancet 2016;387:1540–50. doi:10.1016/S0140-6736(15)01281-7
3. Herbst RS, Baas P, Perez-Gracia JL, et al. Use of archival versus newly collected tumor samples for assessing PD-L1 expression and overall survival: an updated analysis of KEYNOTE-010 trial. Ann Oncol 2019;30:281–9. doi:10.1093/annonc/mdy545

**Keynote-024**

1. Bhadhuri A, Insinga R, Guggisberg P, et al. Cost effectiveness of pembrolizumab vs chemotherapy as first-line treatment for metastatic NSCLC that expresses high levels of PD-L1 in Switzerland. Swiss Med Wkly 2019;149:w20170. doi:10.4414/smw.2019.20170
2. Brahmer JR, Rodríguez-Abreu D, Robinson AG, et al. Health-related quality-of-life results for pembrolizumab versus chemotherapy in advanced, PD-L1-positive NSCLC (KEYNOTE-024): a multicentre, international, randomised, open-label phase 3 trial. Lancet Oncol 2017;18:1600–9. doi:10.1016/S1470-2045(17)30690-3
3. van Vugt MJH, Stone JA, De Greef RHJMM, et al. Immunogenicity of pembrolizumab in patients with advanced tumors. J Immunother Cancer 2019;7:212. doi:10.1186/s40425-019-0663-4
4. Reck M, Rodríguez-Abreu D, Robinson AG, et al. Pembrolizumab versus Chemotherapy for PD-L1-Positive Non-Small-Cell Lung Cancer. N Engl J Med 2016;375:1823–33. doi:10.1056/NEJMoa1606774
